# Addressing the challenges of E-cigarette safety profiling by assessment of pulmonary toxicological response in bronchial and alveolar mucosa models

**DOI:** 10.1038/s41598-020-77452-w

**Published:** 2020-11-24

**Authors:** Koustav Ganguly, Axel Nordström, Tania A. Thimraj, Mizanur Rahman, Malin Ramström, Shanzina I. Sompa, Elizabeth Z. Lin, Fiona O’Brien, Jeremy Koelmel, Lena Ernstgård, Gunnar Johanson, Krystal J. Godri Pollitt, Lena Palmberg, Swapna Upadhyay

**Affiliations:** 1 Unit of Integrative Toxicology, Institute of Environmental Medicine (IMM), Karolinska Institutet, Stockholm, Sweden; 2 Department of Environmental Health Sciences, School of Public Health, Yale University, New Haven, CT USA

**Keywords:** Diseases, Risk factors

## Abstract

Limited toxicity data on electronic cigarette (ECIG) impede evidence-based policy recommendations. We compared two popular mixed fruit flavored ECIG-liquids with and without nicotine aerosolized at 40 W (E-smoke) with respect to particle number concentrations, chemical composition, and response on physiologically relevant human bronchial and alveolar lung mucosa models cultured at air–liquid interface. E-smoke was characterized by significantly increased particle number concentrations with increased wattage (25, 40, and 55 W) and nicotine presence. The chemical composition of E-smoke differed across the two tested flavors in terms of cytotoxic compounds including p-benzoquinone, nicotyrine, and flavoring agents (for example vanillin, ethyl vanillin). Significant differences in the expression of markers for pro-inflammation, oxidative stress, tissue injury/repair, alarm anti-protease, anti-microbial defense, epithelial barrier function, and epigenetic modification were observed between the flavors, nicotine content, and/ or lung models (bronchial or alveolar). Our findings indicate that ECIG toxicity is influenced by combination of multiple factors including flavor, nicotine content, vaping regime, and the region of respiratory tree (bronchial or alveolar). Toxic chemicals and flavoring agents detected in high concentrations in the E-smoke of each flavor warrant independent evaluation for their specific role in imparting toxicity. Therefore, multi-disciplinary approaches are warranted for comprehensive safety profiling of ECIG.

## Introduction

The electronic cigarette (ECIG) market is projected to grow from USD 9.4 to 58.3 billion by 2026^1^. However, the current safety profile of ECIG is insufficient^2^. Lack of long-term toxicity data and systematic risk assessment strategy are the primary hindrances for the regulators to outline evidence-based policy recommendations^3^. ECIG heat (200–350 °C) a liquid solution (E-liquid) until the liquid vaporizes and produces an aerosol (E-smoke), which is inhaled by the ECIG-user. E-liquid is composed of water, propylene glycol, glycerol, flavorings, and with or without nicotine, The E-smoke contains harmful and potentially harmful constituents in the fine and ultrafine particulate matter size fractions, nicotine, flavors such as diacetyl that causes the deadly popcorn lung disease (bronchiolitis obliterans), 2,3-pentanedione, polycyclic aromatic hydrocarbons (PAHs), volatile organic compounds (VOCs), nitrosamines, reactive aldehydes such as formaldehyde and acrolein and heavy metals^3,4^. Hundreds of devices, > 8000 flavors, online retailing, and unregulated and open market makes the scenario challenging. The promotion of ECIG by tobacco industry as a smoking cessation tool remains undetermined^4^. On the contrary, ECIG are considered as a gateway for nicotine addiction among adolescents and youth with its appealing flavors^5–9^. In 2017, 63 million Europeans aged 15 years or older had used ECIG at least once, and 7.6 million were regular ECIG users^10^. The majority of ECIG users in the US are between 18 and 24 years of age^7^. Use of ECIG have surpassed tobacco use among US teenagers^11^.

By January 2020, more than 2700 ECIG, or vaping, product use-associated lung injury (EVALI) cases and 60 EVALI related deaths had been reported in US^12,13^. The emergence of case reports (53 patients with median age 19 years) related to EVALI suggests various symptoms and modes of toxicity^14^. The patients reported respiratory symptoms such as shortness of breath, chest pain, pleuritic chest pain, and cough. Bilateral infiltration in the lung, ground-glass opacification, lipid laden macrophages, mild and non-specific inflammation, acute diffuse alveolar damage with foamy macrophages, and interstitial and peri-bronchial granulomatous pneumonitis were observed^14^.

Inflammatory responses, oxidative stress, cell death, epithelial barrier change, and DNA damage are regarded as major endpoints of ECIG mediated effects^2–4,15–17^. Moreover, studies in mice found that chronic E-smoke exposure downregulate innate immunity in resident macrophages^18^. The cytotoxicity of E-liquids has been attributed to the presence of various flavoring chemicals such as ethyl maltol, furaneol, ethyl vanillin, vanillin, benzyl alcohol, ethyl butanoate, triacetin, acetoin, and ethyl acetate^6^. The recent recommendations of the European Respiratory Society task force on ECIG research include the need for identification of molecular patterns as well as studies characterizing the health effects and toxicology of ECIG flavorings^4^. Limited data are available comparing the composition and biological effect of non-nicotinized (−NIC) and nicotinized (+NIC) E-smoke from the same flavor.

In this study we compared the particle number concentration (PNC), particle size distribution (PSD), chemical composition, and pulmonary molecular effects of two popular mixed fruit flavored E-liquids with and without nicotine (±NIC). Human primary bronchial epithelial cells (PBEC) and representative human type II alveolar cells cultured at air–liquid interface (ALI) were used to develop physiologically relevant bronchial- (bro-ALI) and alveolar mucosa (alv-ALI) models for assessing the molecular effects of E-smoke exposure.

## Methods

Briefly, the materials and methods are described here with detailed description in the supplementary section.

### E-smoke generation

Third generation electronic nicotine delivery systems with refillable and exchangeable tank options were used to generate the E-smoke. Two popular sweet mixed fruit flavored E-liquids (ECIG-flavor-1: raspberry, orange, lemon and lime; ECIG-flavor-2: ripe strawberry, sweet apples and tart kiwi; ±NIC for both flavors) were used for experimental purposes. The lowest nicotine concentration (3 mg/mL) available on the Swedish market was used. A vaping regime mimicking a low intensity vaping was considered for short-term repeated exposure for one day [40 watts (W), 40 ml/puff, 3 seconds (s) puff duration, 30 s puff interval, 10 puffs/session, bro-ALI model: 6 sessions/day, alv-ALI model: 3 sessions/day, 1 h session interval] leading to a total of 60 puffs and 30 puffs for bro-ALI and alv-ALI, respectively. The exposure of the alv-ALI model was shortened since 60 puffs exposure was excessively cytotoxic for them as detected in pilot studies. Sham were exposed to clean air under identical conditions and served as control. The vaping regime was elaborated based on the available literature^19–23^. We also exposed both bro-ALI and alv-ALI models under the same conditions (3 vaping sessions each, i.e. 30 puffs in total) of ECIG-flavor-2 and measured total reactive oxygen species (ROS) generation in the cells using flow cytometry.

### Particle number concentration and size distribution

A portable laser spectrometer [model Mini-LAS 11R; GRIMM, Aerosol Technik GmbH and Co. KG, Germany) was used for measuring the PNC and PSD (instrument range 0.25–32 µm) of the E-smoke from both ECIG-flavors-1 and 2 (±NIC). E-smoke was generated at three different power settings (25, 40 and 55 W representing low, medium and high power) for measuring the PNC and PSD.

### Chemical characterization of E-smoke

Twelve compounds commonly reported to be present in E-smoke^24–26^ were screened using gas chromatography with flame ionization detection following aerosolization/combustion (at 40 W for 3 s) of the ECIG-flavor-1(±NIC) as a preliminary screen. The selected compounds were: 1-pentanol, 2,3-pentadione, acetoin, acetic acid, acetone, acrolein, crotonaldehyde, diacetyl, methanol, nicotine, propionalaldehyde, and toluene. The peaks in the aerosolized samples were compared to chromatograms of reference compounds for detection.

For both suspect screening and quantitative analysis of select analytes of E-smoke (aerosolized at 40 W for 3 s), a gas chromatography Q-Exactive mass spectrometer was used in electron ionization mode. Briefly, Thermos Deconvolution Plugin (Thermo Fisher Scientific, Waltham, MA) was used for suspect screening using the NIST 17 and Thermo spectral libraries. The data was deconvoluted, peaks were identified with conservative filtering criteria for higher confidence in results (Kovat's retention index, reverse dot product, and high mass accuracy filters), aligned, and blank filtered. Relevant sources/categories of the identified compounds in the final annotated dataset were determined using the chemical and products database (CPDat)^27^. In addition to suspect screening, targeted analysis of 92 compounds including pesticides, phthalates, and poly-aromatic hydrocarbons were semi-quantified using external calibration and 7 internal standards. Blank filtering was applied as in suspect screening. The list of compounds is provided in the supplementary section (Excel file).

### Bronchial and alveolar mucosa models

#### Bronchial

The bro-ALI model was developed using PBEC from 3 to 4 donors (N) with 3 technical replicates (n) from each donor. The PBEC were harvested from healthy bronchial tissues obtained from donors in connection with lobectomy following written and informed consent, and approval by the Swedish Ethical Review Authority (Institutional ethic committee reference number 99-357). The detailed protocol and details of cellular differentiation (club cells, goblet cells, basal cells, ciliated cells, etc.) of the bro-ALI model have been described previously^28,29^. The cells used in this study are well characterized and have been used in connection with several other projects^28–34^. All experiments and methods were carried out in accordance with relevant guidelines and regulations.

#### Alveolar

The NCI-H441 (ATCC HTB-174) cell line, known to express constitutively the mRNA and protein of the major surfactant apo-protein (SP-A) was used to develop the alv-ALI model. NCI-H441 cells were co-cultured with HULEC-5a (ATCC CRL-3244) representative of human lung microvascular endothelial cells for this purpose. NCI-H441 cells (passages 51–53; and 2 technical replicates of each) were cultured on separate Petri dishes (ThermoFisher Scientific, Massachusetts, USA) precoated with coating buffer fibronectin (1 mg/ml, Gibco, UK), bovine serum albumin fraction V (BSA; 1 mg/ml; Sigma, Germany), vitrogen 100 collagen (3.1 mg/ml; Cohesion Technologies, USA) and PBS without Ca^2+^/Mg^2+^ (Life technologies, Paisley, UK) using OptiMEM medium (Gibco: 31985047) supplemented with 10% FBS (Gibco: 10082147) and penicillin streptomycin antibiotics (PEST, 1%; Gibco, UK: 15140122, 100 U/100 μg/ml) at 37 °C and 5% CO_2_. HULEC-5a (passage 23) were maintained on pre-coated (fibronectin and collagen) T75 flasks using M199 medium supplemented with 15% FBS, 2 mM Glutamax I (Sigma, Germany: 35050061), 25 μg/ml sodium heparin (Sigma, Germany; H3149-10KU), 25 μg/ml endothelial cell growth supplements (Sigma, Germany: 211-GS SIGMA), and PEST (1%) at 37 °C and 5% CO_2_.

To build the alveolar models, NCI-H441 cells were cultured on pre-coated semi-porous (0.4 µm diameter) transwell inserts (BD Falcon, USA) in 12-well plates with a seeding density of 250,000 cells/cm^2^. After attaining confluency around day 7, the inserts were turned upside down and placed into a sterile Petri dish and HULEC-5a cells (9 × 10^4 ^cells/cm^2^) were added on the lower surface of transwell to develop co-culture system. The Petri dish was covered and incubated at 37 °C for 30 minutes (min). HULEC-5a maintenance medium (50 µl M199 with supplements: complete M199) was added every 10 min to keep the cells humid after 30 min of incubation. After a total of 1 h incubation, the inserts were placed back into the plates and 1 ml M199 complete medium both at apical and basal side of each inserts were added. After overnight incubation at 37 °C with 5% CO_2_, models were airlifted by removing the medium from apical side and adding 1 ml of complete M199 medium with 1 μM Dexamethasone (Sigma: D4902) only at the basal side of the inserts. To induce differentiation of the NCI-H441 cell line in co-cultures, 1 μM Dexamethasone was added in the ALI medium (complete M199). Light-, confocal microscopy and transepithelial electrical resistance (TEER) measurement was used to characterize the morphology of differentiated H441 at ALI condition. Quantitative real-time polymerase chain reaction (qRT-PCR) was performed to assess the expression of alveolar type I (ATI: aquaporin 5/AQP5) and type II [ATII: surfactant protein A (SPA), SPB, SPC] specific markers. In addition, cell viability assay (Trypan blue staining), apoptosis assay (annexin V), histological analysis (Hematoxylin and Eosin staining), confocal microscopy [for immunofluorescence detection of zona occludin 1 (ZO1) alternatively known as tight junction protein 1 (TJP1), SP-C, LysoTracker Green DND-25, epithelial sodium channel (ENaC)] and transmission electron microscopy (as described previously^28^) was performed to characterize the alv-ALI model. Transcript expression of representative tight junction proteins *TJP1* and claudins (*CLDN 5, 7*) have been assessed in the alveolar model following E-smoke exposure of both flavors (±NIC).

### E-smoke exposure system

**Figure 1:**
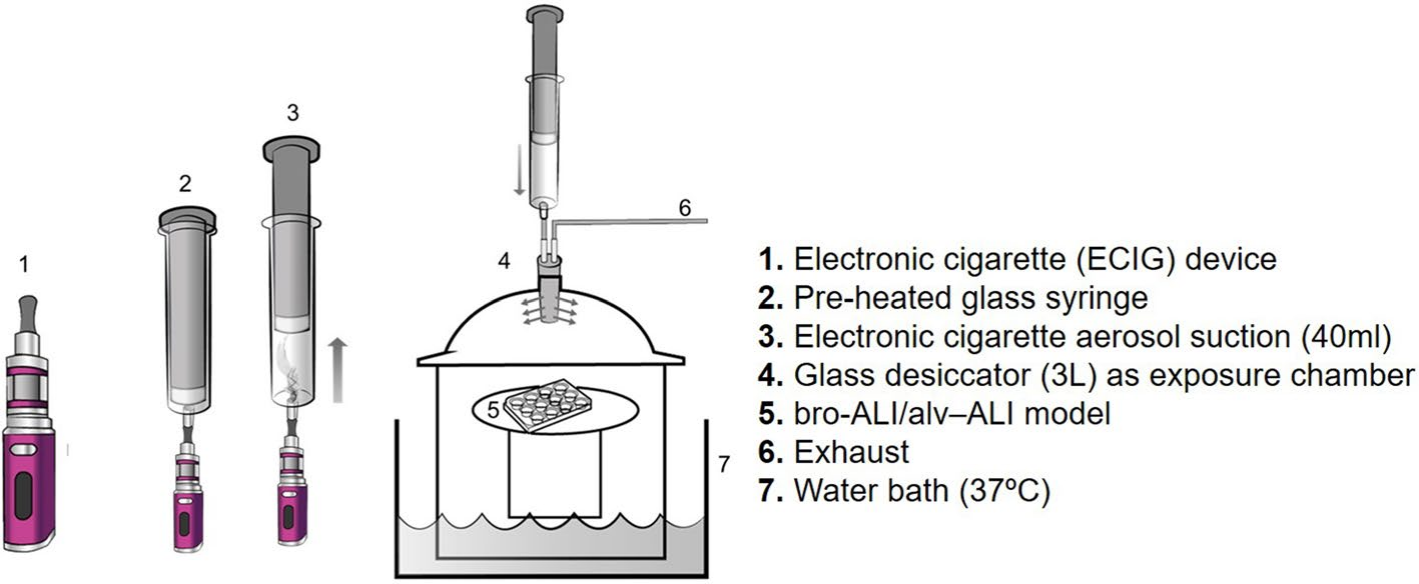
Schematic representation of the electronic cigarette (ECIG) aerosol exposure set up for bronchial (bro-ALI) and alveolar (alv-ALI) mucosa models. A vaping regime mimicking one day of low intensity vaping was applied and consisted of repeated exposure [40 watts (W), 40 ml/puff, 3 s (s) puff duration, 30 s puff interval, 10 puffs/session, bro-ALI model: 6 sessions, alv-ALI model: 3 sessions, 1 h session interval] leading to a total of 60 puffs and 30 puffs for bro-ALI and alv-ALI, respectively. The authors sincerely acknowledge the assistance of Ann-Katrin Sjödén in preparing the figure.

A schematic representation of the E-smoke exposure set up is provided in Fig. 1. In brief, the bro-ALI and alv-ALI models were developed in transwell inserts in 12-well plates. After change of cell medium, the plates were placed in a 3L desiccator glass jar maintained at 37 °C and 60% humidity and allowed to equilibrate for 5 min. The volume of the desiccator represents the functional residual capacity of a healthy adult human lung^35^. An air-tight pre-heated glass syringe was used to repeatedly collect 40 ml (representing one puff) of E-smoke and inject into the desiccator. Ten puffs were injected to mimic one vaping session. The inlet tube contained multiple sidewise apertures for even spread of the E-smoke within the desiccator. The lung models were exposed to E-smoke or clean air for 15 min, where after they were transferred to a cell incubator (37 °C, 60% humidity and 5% CO_2_) for 1 hour (h) until next exposure session. Following completion of repeated exposures, the models were incubated for 24 h prior to collection of basal media and cell inserts. None of the exposure regimes induced cytotoxicity as assessed by lactate dehydrogenase for the bro-ALI (LDH; cat# 88953; Thermo Fisher scientific) and propidium iodide staining (cat#556463; BD bioscience) for the alv-ALI models (data not shown). Propidium iodide staining was used for NCI-H441 cells due to their high basal secretion of LDH.

### Assessment of E-smoke related pulmonary molecular response

#### Gene expression

The mRNA expression levels of markers for proinflammation: C-X-C motif chemokine ligand 8 *(CXCL8),* interleukin (*IL)1B, IL6,* nuclear factor kappa B subunit 1 *(NFKB1)* and tumor necrosis factor *(TNF*); oxidative stress: glutathione S-transferase alpha 1 *(GSTA1),* heme oxygenase 1 *(HMOX1),* and superoxide dismutase 3, extracellular *(SOD3*); tissue injury/repair: matrix metallopeptidase 9 (*MMP9*), and TIMP metallopeptidase inhibitor 1 (*TIMP1*), alarm anti-proteases: peptidase inhibitor 3 [*PI3*, alternatively known as elafin], and secretory leukocyte peptidase inhibitor (*SLPI*), and anti-microbial defense response: defensin beta 4A (*DEFB4A,* alternatively known as defensin 2) were measured in the two lung models using qRT-PCR (ΔΔC_T_ method). Actin beta (*ACTB*) was used as the reference gene as described previously^30–33^. Transcript expression of secretoglobin family 1A member 1 (*SCGB1A1,* alternatively known as club cell protein 10) and mucin 5AC, oligomeric mucus/gel-forming (*MUC5AC*) were also assessed as markers of club and goblet cells in the bro-ALI model respectively. Expression of DNA methyl transferase (*DNMT1,3A*, and *3B*) genes have been also assessed. Primer pair sequences of the investigated genes not previously reported (*AQP5, CLDN5,7; DEFB4A, DNMT1, DNMT3A, DNMT3B, PI3, SP-A,B,C,D; TJP1*) are provided in the Supplementary Table ST1. A two-fold increase or decrease in transcript expression level was set as cut off along with statistical significance.

#### Protein concentration

Secreted protein levels of DEFB4A (EKH1674 , Nordic Biosite), PI3 (DY1747, R&D Systems), SCGB1A1 (DY4218, R&D Systems), and SLPI (DY1274-05, R&D Systems) in the basal media of bro-ALI (N = 3–4; n = 3) and alv-ALI (3 passages; 2 replicates/passage) were measured following E-smoke (±NIC) exposure by ELISA according to manufacturer instruction. Concentrations of cytokines CXCL8, IL1B, IL6, IL10, IL13, and TNF were also measured in the basal media of bro-ALI (N = 3; n = 3) and alv-ALI (3 passages; 2 replicates/passage) following E-smoke (±NIC) exposure using the V-plex immunoassay platform of Meso Scale Discovery Inc (Rockville MD) at the Clinical Biomarkers facility, Science for Life Laboratory, Uppsala University, Sweden.

#### DNA methylation and hydroxymethylation

To assess the plausible epigenetic effect of E-smoke exposure, DNA methylation (ab117128, Abcam) and hydroxymethylation (ab117130, Abcam) was measured in the alv-ALI model (3 passages: 2 replicates/passage) according to manufacturer instruction. Total genomic DNA was isolated using Abcam genomic DNA Isolation Kit (ab65358) according to manufacturer instruction. 75 ng (ECIG flavor-1)/100 ng (ECIG flavor-2) and 150 ng total DNA was used for methylation and hydroxymethylation assays respectively and compared to corresponding sham. Data are represented as percentage of 5-methylcytosine (**5-mC**) or 5-hydroxymethylcytosine (**5-hmC**) in total DNA. Expression of *DNMT1, DNMT3A,* and *DNMT3B* have been also assessed. Only the alv-ALI model was used for this assay as our ethical permit did not include DNA analysis from PBEC.

### Statistics

The results (gene and protein expression, methylation and hydroxymethylation) are expressed as median and interquartile ranges (25th–75th percentiles) followed by non-parametric statistical analysis^28,32^. Within each group (bro-ALI and alv-ALI models), the comparisons between ECIG-flavors (±NIC) and corresponding sham were assessed by Friedman test and followed by Wilcoxon signed rank t test as a post hoc test. Since bro-ALI and alv-ALI lung mucosa models were exposed to different doses of E-smoke, no statistical comparisons were performed between them. For, normally distributed data (i.e. PNC), one-way ANOVA followed by t-test was performed. In all tests, difference with p values below 0.05 were considered significant. All the data were analyzed using the STATISTICA9 software (StatSoft, Inc. Uppsala, Sweden). Only significantly different data are mentioned in the results section.

## Results

### Particle number concentrations and size distribution versus nicotine content, power setting, and flavor

For both ECIG flavors, the PNC increased with the applied wattage (Supplementary Figure S1 and Supplementary Table ST2). In addition, the presence of nicotine (+NIC) generally resulted in increased particle counts, the one exception being ECIG-flavor-1 at 40 W. The two flavors showed similar PSD with sizes ranging between 0.25–3.5 µm with the peak mode diameter around 0.58 µm (Supplementary Figure S2).

### Chemical composition

Analysis of ECIG-flavor-1 (+NIC) by gas chromatography with flame ionization detected 1-pentanol, 2,3-pentadione, acetoin, acetic acid, acetone, acrolein, crotonaldehyde, diacetyl, methanol, propionaldehyde and toluene along with nicotine (Supplementary table ST3). Corresponding analysis of ECIG-flavor-1 (−NIC) detected 1-pentanol, 2,3-pentadione, acetic acid, acetone, acrolein, diacetyl, methanol, and toluene (Supplementary table ST3).

**Figure 2:**
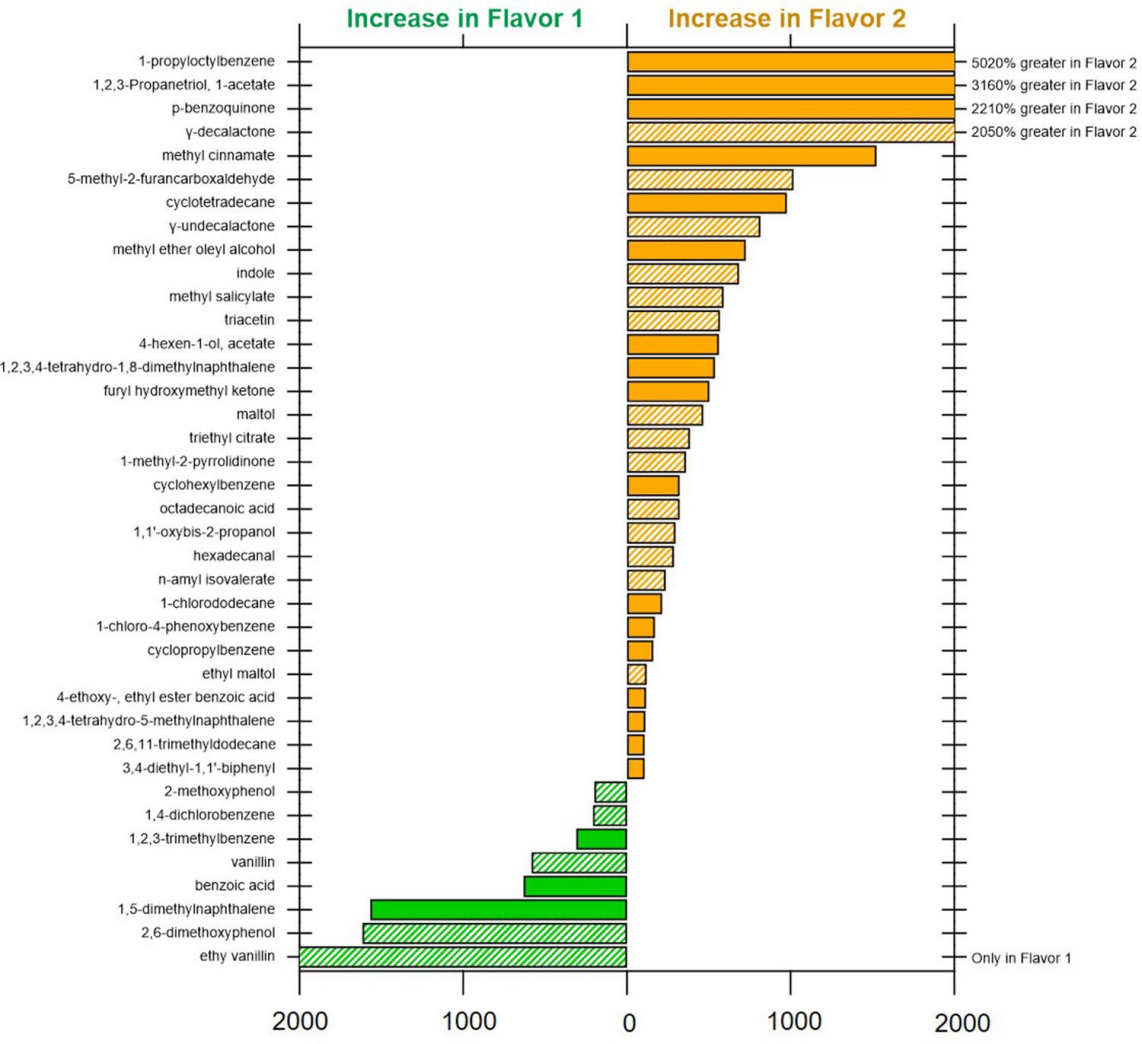
Percent difference in concentration of the top 40 identified compounds in suspect screening by gas chromatography–mass spectrometry comparing electronic cigarette (ECIG) aerosol of ECIG-flavor-1 and ECIG-flavor-2 (both without nicotine) generated at 40 W. All compounds with twofold difference between flavors included. Shaded bars indicate flavor or fragrance-related compounds.

In the gas chromotaography Q-Exactive mass spectrometer-based screening of ECIG-flavor-1 and ECIG-flavor-2 (±NIC), 92 unique compounds were annotated using stringent filters. A list of identified chemicals, their signal intensities, and reported sources are provided in the supplementary Excel file. Of the 92 compounds identified, only 48 had known sources in the CPDat database^27^. Of these, 67% were compounds categorized as being flavoring agents or fragrances for consumer use. These include maltol, ethyl maltol, ethyl vanillin, vanillin, and furaneol. The remaining 33% and compounds without source information were predominantly aromatic hydrocarbons (Supplementary Excel file). A comparison of measurements of the top 40 compounds across ECIG-flavors-1 and 2 is shown in Fig. 2. Other compounds of interest include p-benzoquinone which was one of the topmost abundant signals detected and nicotyrine. ECIG-flavor-1 did have a higher levels of top cytotoxic compounds vanillin and ethyl vanillin (not detected in ECIG-flavor-2). Of the 92 compounds screened in the targeted approach, 11 were detected above the blank signal threshold, including triphenyl phosphate, tris(1-chloro-2-propyl) phosphate, nicotine, 4-chloroaniline, benzene, 1-chloro-4-phenoxy, benzene, 1,3-dichloro-, benzene, 1,4 dichloro-, benzyl alcohol, butylbenzyl phthalate, n-nitrosodiphenylamine, nitrobenzene. Elevated levels of compounds detected by previously described gas chromatography with flame ionization which included high levels of expected compounds (e.g. benzyl alcohol and nicotine) were similarly found by this suspect screening analysis. Relative quantities can be found in the Supplementary Excel file. It would be of interest to analyze the change in composition of E-smoke of these flavors generated at different wattage/power settings of the ECIG device and to know how this is correlated to toxicity.

### Bronchial model

**Figure 3:**
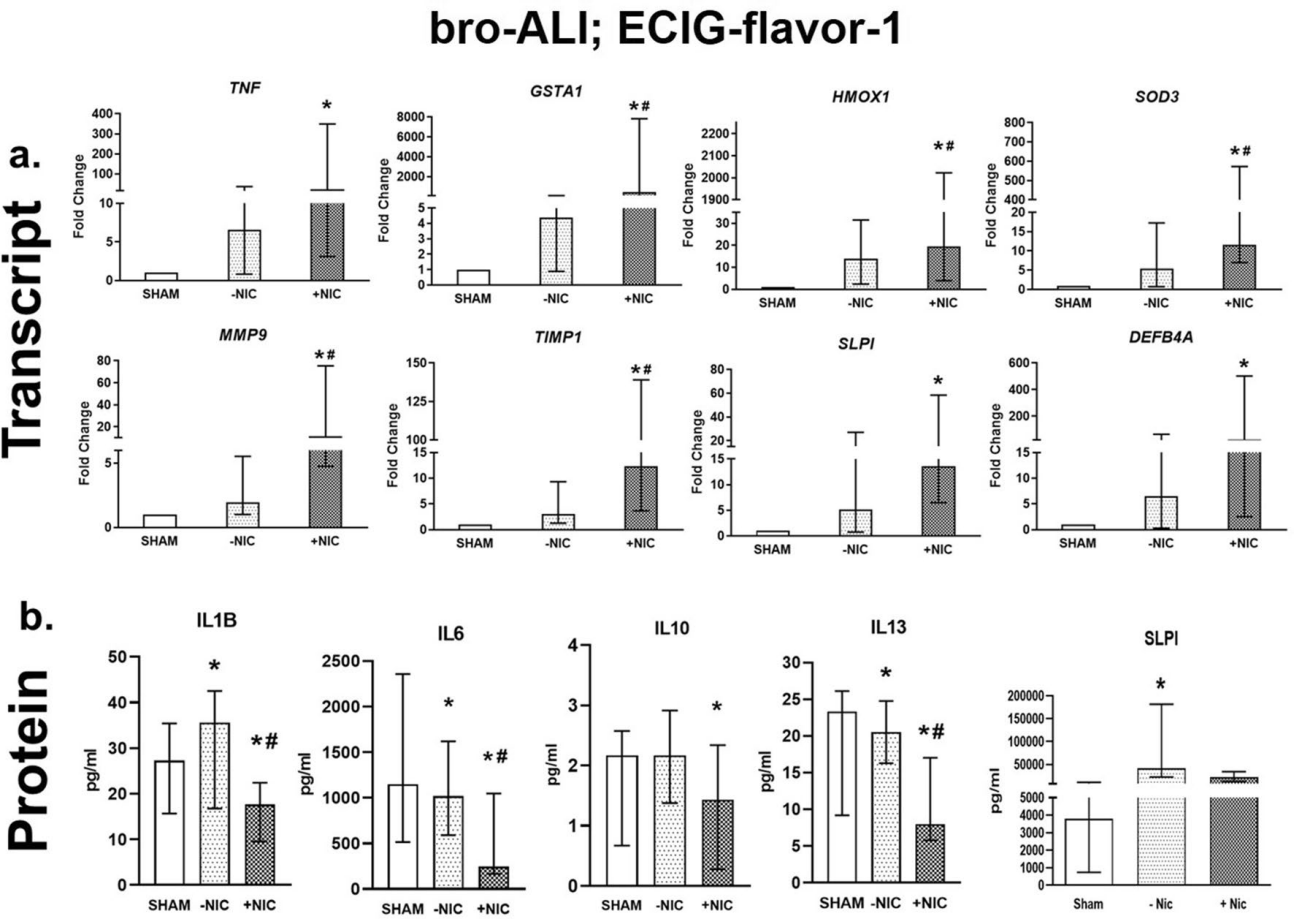
Transcript expression (**a**) and secreted protein levels (**b**) of significantly altered pro-inflammatory, oxidative stress, tissue injury/repair, alarm anti-proteases, and/ or anti-microbial defensin markers in the bronchial mucosa model cultured at air–liquid interface (bro-ALI) following exposure to aerosolized non-nicotinized (−NIC) and nicotinized (+NIC) electronic cigarette liquid flavor 1 (ECIG-flavor-1). Actin beta (*ACTB*) was used as the reference gene. Fold changes for transcript expression were calculated relative to the corresponding sham. *: significantly different from sham; #: significantly different from −NIC (*p* < 0.05, Friedman followed by Wilcoxon test). **bro-ALI:** bronchial mucosa model developed at air–liquid interface **DEFB4A**: defensin beta 4A, **GSTA1**: glutathione S-transferase alpha 1, **HMOX1**: heme oxygenase 1, **IL:** interleukin, **MMP9**: matrix metallopeptidase 9, **SLPI**: secretory leukocyte peptidase inhibitor, **SOD3**: superoxide dismutase 3, extracellular, **TIMP1**: TIMP metallopeptidase inhibitor 1, **TNF**: tumor necrosis factor.

Exposure of ECIG-flavor-1(−NIC) to bro-ALI did not result in significant alteration of any of the investigated markers at transcript level representing the molecular biological pathways of pro-inflammation, oxidative stress, tissue injury/repair, alarm anti-proteases and anti-microbial defense response (Fig. 3a). In contrast, ECIG-flavor-1 (+NIC) caused increased *TNF* (> 22-fold), *GSTA1* (> 450-fold), *HMOX1* (> 20-fold), *SOD3* (> 11-fold), *MMP9* (> 11-fold), *TIMP1* (> 12-fold), *SLPI* (> 13-fold), and *DEFB4A* (> 22-fold) (Fig. 3a). The expression of bronchial cell specific markers *MUC5AC* and *SCGB1A1* were unaffected by ECIG-flavor-1 (±NIC). At 24 h post exposure, secreted levels of IL1B, IL6, IL10 and IL13 were reduced in the ECIG-flavor-1 (+NIC) in the bro-ALI model (Fig. 3b). In the ECIG-flavor-1 (−NIC) bro-ALI model, IL1B and SLPI were increased whereas IL6 and IL13 were slightly reduced (Fig. 3b).

**Figure 4:**
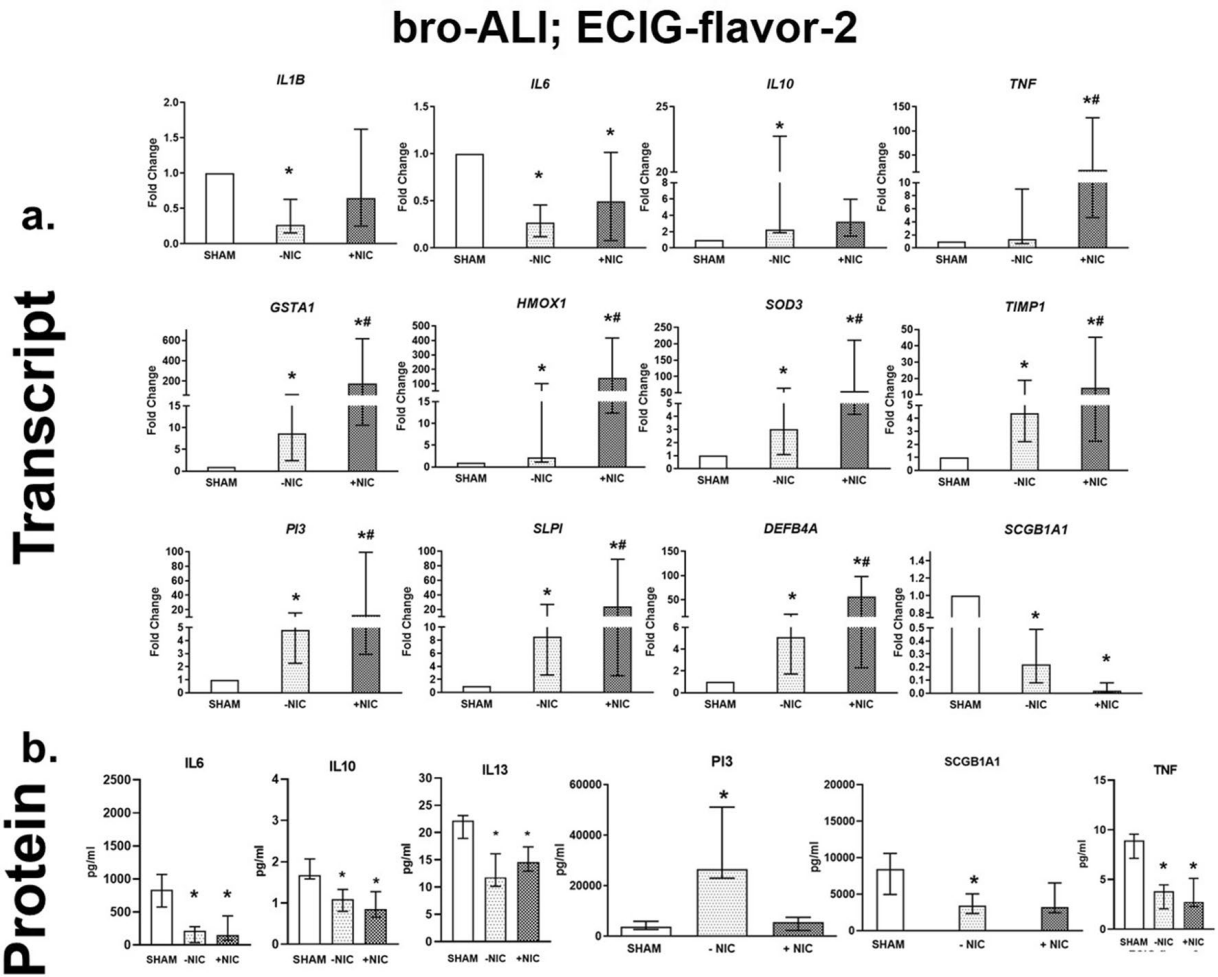
Transcript expression (**a**) and secreted protein levels (**b**) of significantly altered pro-inflammatory, oxidative stress, tissue injury/repair, alarm anti-proteases, and/ or anti-microbial defensin markers in the bronchial mucosa model cultured at air–liquid interface (bro-ALI) following exposure to aerosolized non-nicotinized (−NIC) and nicotinized (+NIC) electronic cigarette liquid flavor 2 (ECIG-flavor-2). Actin beta (*ACTB*) was used as the reference gene. Fold changes for transcript expression were calculated relative to the corresponding sham *: significantly different from sham; #: significantly different from −NIC (*p* < 0.05, Friedman followed by Wilcoxon test). **DEFB4A**: defensin beta 4A, **GSTA1**: glutathione S-transferase alpha 1, **HMOX1**: heme oxygenase 1, **IL:** interleukin, **PI3**: peptidase inhibitor 3, ***SCGB1A1***: secretoglobin family 1A member 1, **SLPI**: secretory leukocyte peptidase inhibitor, **SOD3**: superoxide dismutase 3, extracellular, **TIMP1**: TIMP metallopeptidase inhibitor 1, **TNF**: tumor necrosis factor.

ECIG-flavor-2 (−NIC) exposure resulted in decreased *IL1B* (> threefold), *IL6* (> threefold), and increased *IL10* (> twofold) amongst the markers of proinflammation (Fig. 4a). Oxidative stress markers *GSTA1* (eightfold), *HMOX1* (> twofold) and *SOD3* (> threefold) were increased on exposure to ECIG-flavor-2 (−NIC) (Fig. 4a). Increased expression of anti-protease *TIMP1* (> fourfold), alarm anti-proteases *SLPI* (> fivefold) and *PI3* (> fourfold), and anti-microbial defensin *DEFB4A* (> fivefold) following ECIG-flavor-2 (−NIC) exposure was detected (Fig. 4a). ECIG-flavor-2 (+ NIC) caused a > two-fold decreased *IL6* levels (Fig. 4a). Increased levels of *TNF* (> 19-fold), *HMOX1* (> 140-fold), *GSTA1* (> 176-fold), *SOD3* (> 55-fold), *TIMP1* (> 14-fold), *SLPI* (> 56-fold), *PI3* (> 12-fold), and *DEFB4A* (> 56-fold) (Fig. 4a) were detected after exposure to ECIG-flavor-2 (+NIC). The expression of club cell specific protein *SCGB1A1* was reduced by fivefold and 50-fold after exposure to ECIG-flavor-2 (−NIC) and (+NIC) respectively (Fig. 4a). In the bronchial model exposed to ECIG-flavor-2 (±NIC), IL6, IL10. IL13, and TNF levels were reduced (Fig. 4b). Secreted levels of PI3 was increased in case of ECIG-flavor-2 (−NIC). SCGB1A1 concentration was decreased in ECIG-flavor-2 (−NIC) exposed bro-ALI (Fig. 4b).

### Alveolar model

**Figure 5:**
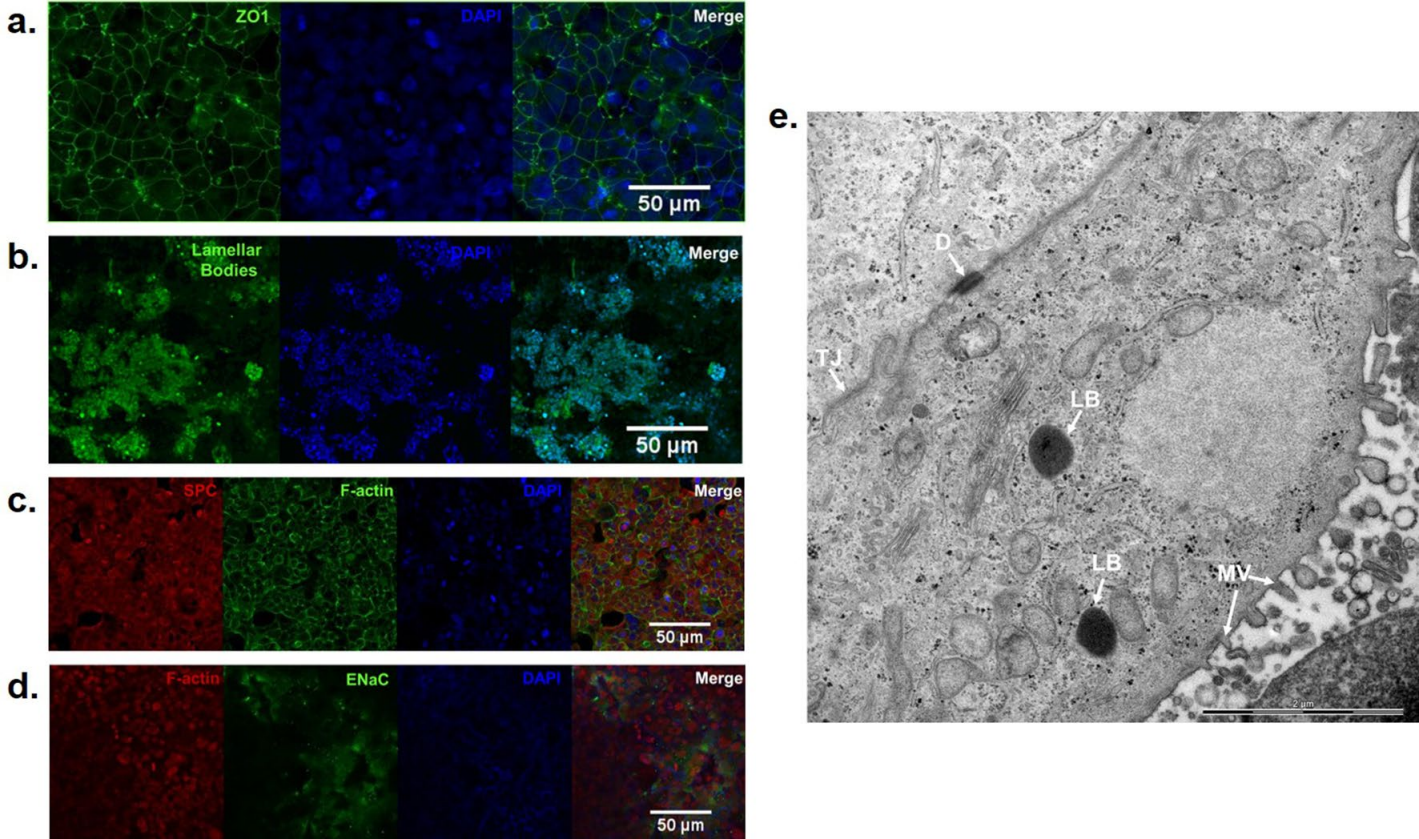
Morphological characterization of the alveolar mucosa model by confocal and transmission electron microscopy (TEM). (**a**) The cell junction protein zona occludin 1 (ZO1) (**b**) lamellar bodies, (**c**) surfactant protein C (SPC) and (**d**) epithelial sodium channel (ENaC). Nucleus is stained in blue. Bar scale: 50 µm; (**e**) Representative TEM image of the alveolar type II cells in air–liquid interface (2 weeks) showing microvilli (MV), lipid bodies (LB), desmosome (D), and tight junction (TJ) Bar scale: 2 µm. The microscopic images are representative of alveolar mucosa model developed at air–liquid interface (2 weeks) from NCl-H441 cells.

Supplementary Figure S3a demonstrates the increasing tight junction potential or epithelial cell barrier integrity (TEER value) of the alv-ALI model from 1 day to week 2. Expression of cell junction protein ZO1 in the alv-ALI model after 2 week is shown in Fig. 5a. The alveolar type II cells in the alv-ALI models were further characterized by the expression of lamellar bodies (Fig. 5b), SPC (Fig. 5c) and ENaC (Fig. 5d). Appearance of co-cultured alv-ALI model (2 weeks) where NCI-H441 cells were cultured on the apical side and HULEC-5a on the basal side of the inserts is demonstrated by H&E staining of model cross sections (Supplementary Figure S3b). Increased *SPA* and *SPC* expression have been detected between 1 day to 2 weeks of alv-ALI model development whereas expression of *SPB* and *AQP5* remained unaltered (Supplementary Figure S3c-f). Transmission electron microscopy of alv-ALI (2 week) shows the presence of microvilli, lipid bodies (as representative of surfactants), desmosomes, and tight junctions as representative characteristics of type II pneumocytes (Fig. 5e).

**Figure 6:**
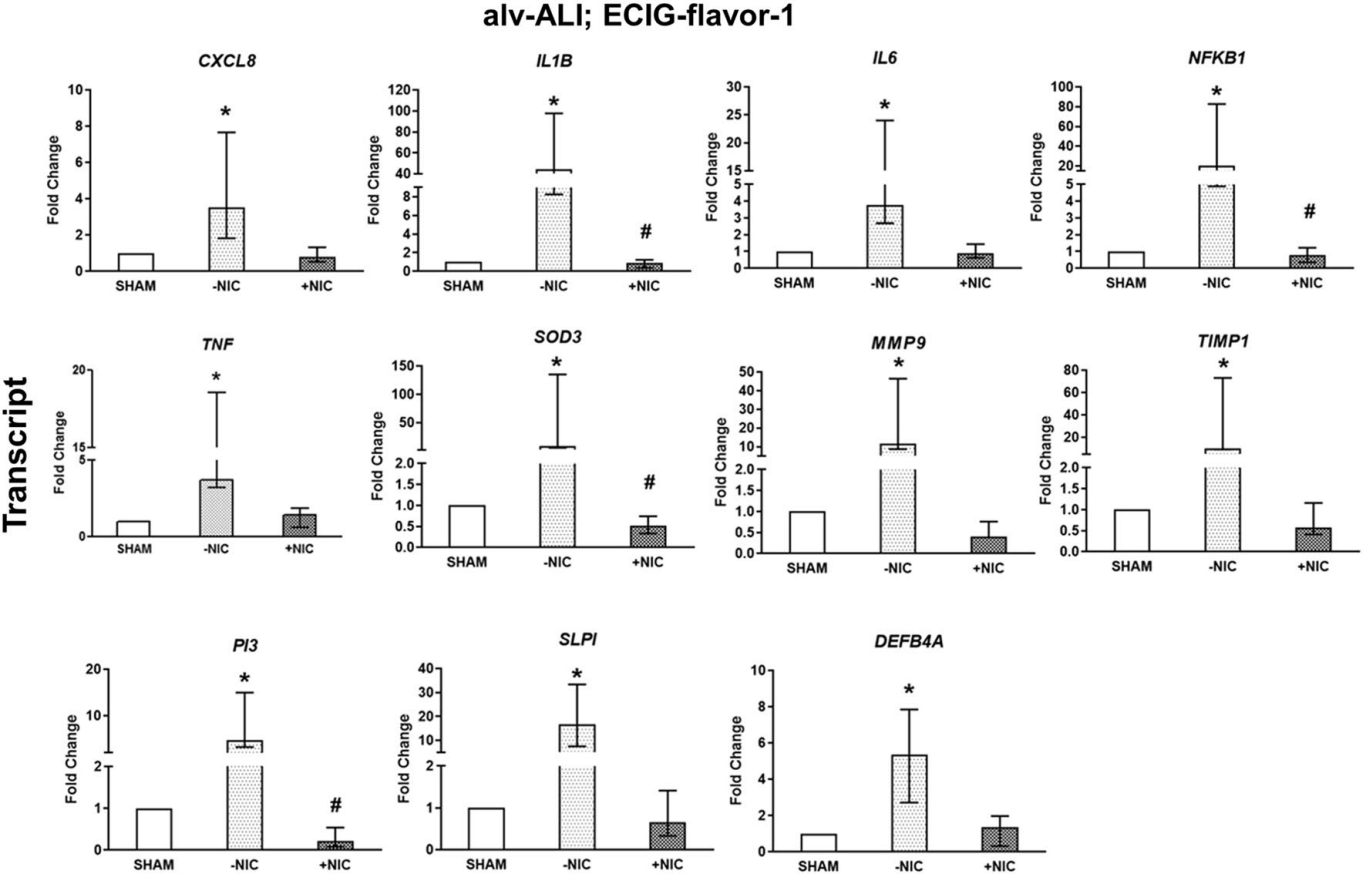
Transcript expression analysis of significantly altered pro-inflammatory, oxidative stress, tissue injury/repair, alarm anti-proteases, and anti-microbial defensin markers in the alveolar mucosa model cultured at air–liquid interface (alv-ALI) following exposure to aerosolized non-nicotinized (−NIC) and nicotinized (+NIC) electronic cigarette liquid flavor 1 (ECIG-flavor-1). Secreted levels of none of the proteins were altered significantly in this exposure condition. Actin beta (*ACTB*) was used as the reference gene. Fold changes for transcript expression were calculated relative to the corresponding sham. *: significantly different from sham; #: significantly different from −NIC (*p* < 0.05, Friedman followed by Wilcoxon test). **CXCL8:** C-X-C motif chemokine ligand 8, **DEFB4A**: defensin beta 4A, **IL:** interleukin, **MMP9**: matrix metallopeptidase 9, **NFKB1:** nuclear factor kappa B subunit 1, **PI3**: peptidase inhibitor 3, **SLPI**: secretory leukocyte peptidase inhibitor, **SOD3**: superoxide dismutase 3, extracellular, **TIMP1**: TIMP metallopeptidase inhibitor 1, **TNF**: tumor necrosis factor.

Exposure to ECIG-flavor-1 (−NIC) to alv-ALI model resulted in increased transcript expression of pro-inflammatory cytokines *CXCL8, IL6, NFKB1,* and *TNF* by more than three-fold whereas *IL1B* was increased by 44-fold (Fig. 6). Oxidative stress response marker *SOD3 levels were increased by* eight-fold (Fig. 6). Concomitant increase of tissue Injury/repair markers MMP*9* (11-fold) and *TIMP1* (ninefold) was also detected (Fig. 6). The alarm anti-proteases *SLPI* was increased by more than 16-fold, PI3 by more than fivefold, and the anti-microbial defense response protein *DEFB4A* by five-fold (Fig. 6). Exposure to ECIG-flavor-1 (+NIC) resulted in down regulation of *IL1B, NFKB1, SOD3,* and *PI3* compared to ECIG-flavor-1 (−NIC) but none of the markers were significantly different to sham (Fig. 6). Secreted levels of none of the protein markers were differentially regulated in alv-ALI exposed to ECIG-flavor-1 (±NIC).

**Figure 7:**
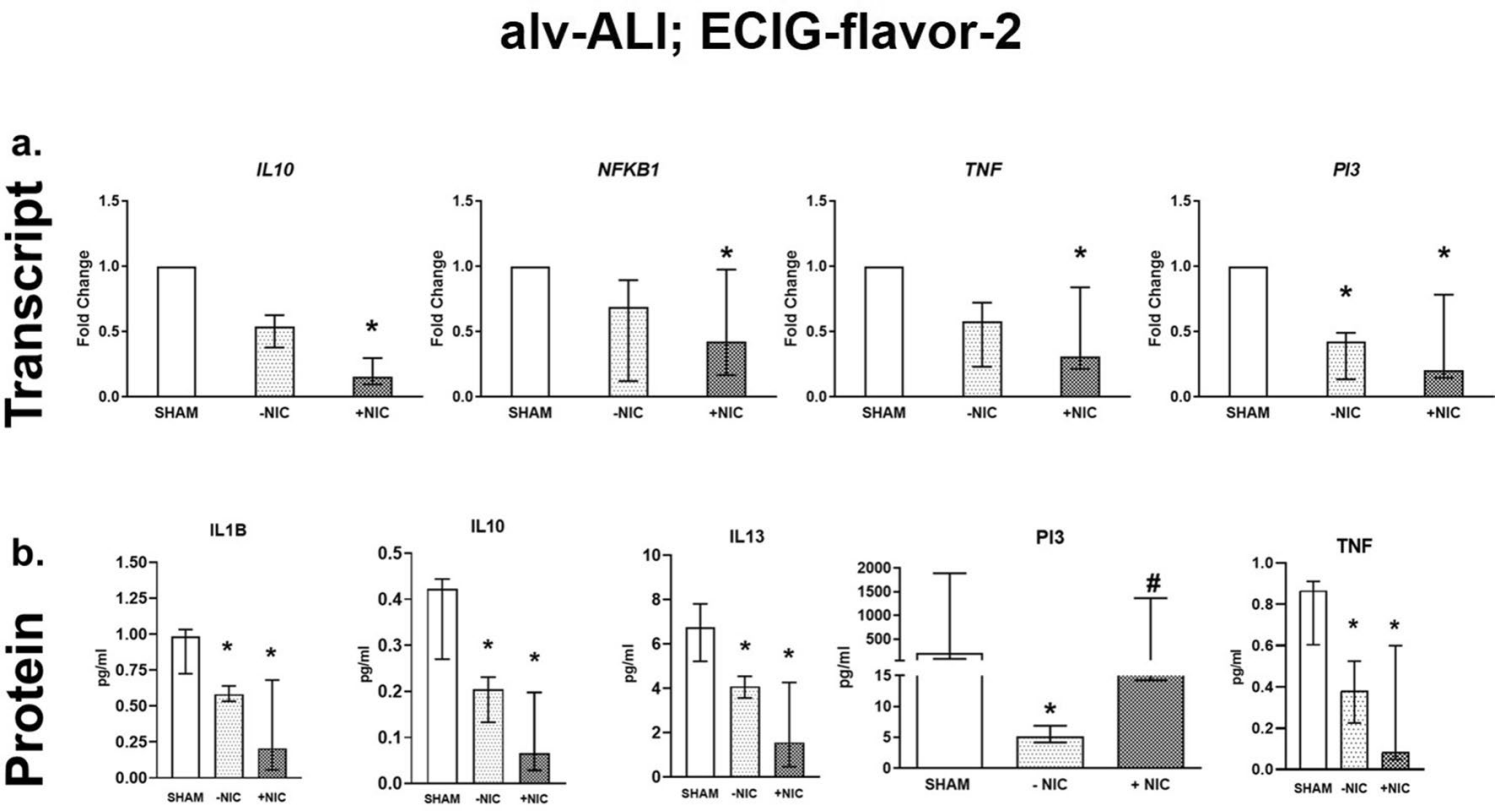
Transcript expression (**a**) and secreted protein levels (**b**) of significantly altered pro-inflammatory, oxidative stress, tissue injury/repair, alarm anti-proteases, and/ or anti-microbial defensin markers in the alveolar mucosa model cultured at air–liquid interface (alv-ALI) following exposure to aerosolized non-nicotinized (−NIC) and nicotinized (+NIC) electronic cigarette liquid flavor 2 (ECIG-flavor-2). Actin beta (*ACTB*) was used as the reference gene. Fold changes for transcript expression were calculated relative to the corresponding sham *: significantly different from sham; #: significantly different from −NIC (*p* < 0.05, Friedman followed by Wilcoxon test). **IL:** interleukin, **NFKB1:** nuclear factor kappa B subunit 1, **PI3**: peptidase inhibitor 3, **TNF**: tumor necrosis factor.

Regarding ECIG-flavor-2 (−NIC) exposure to alv-ALI, expression of only *PI3* was reduced by two-fold (Fig. 7a). Exposure to ECIG-flavor-2 (+NIC) also caused down regulation of pro-inflammatory *NFKB1* (twofold) and TNF (fourfold), anti-inflammatory *IL10* (sixfold), alarm anti-protease *PI3* (fivefold) (Fig. 7a). Furthermore, ECIG-flavor-1 (+NIC) resulted in decreased *SPA* whereas ECIG-flavor-2 (−NIC) (supplementary figure S4a) resulted in decreased *SPB* (supplementary figure S4b). In case of alv-ALI exposed to ECIG-flavor-2 (±NIC), secreted levels of IL1B, IL10, IL13, and TNF were reduced (Fig. 7b). Levels of PI3 was reduced in ECIG-flavor-2 (−NIC) exposed alv-ALI. As an indication of altered barrier function, *TJP1* was increased (thirty-40-fold) in alv-ALI model exposed to both ECIG-flavors-1 and 2 (+NIC) (Supplementary figure S5a, b). Expression of other barrier function markers *CLDN5* and *CLDN7* remained unaltered.

When both the bro-ALI and alv-ALI models were exposed to same exposure conditions (3 vaping sessions each, i.e. 30 puffs in total) of ECIG-flavor-2, significantly increased (twofold) total ROS was detected in both −NIC and +NIC compared to sham in bro-ALI. In case of alv-ALI model, the increase was not statistically significant (Supplementary figure S6a, b). The findings are consistent with the transcript alteration of oxidative stress markers 24 h post-exposure.

### Methylation and hydroxymethylation

**Figure 8:**
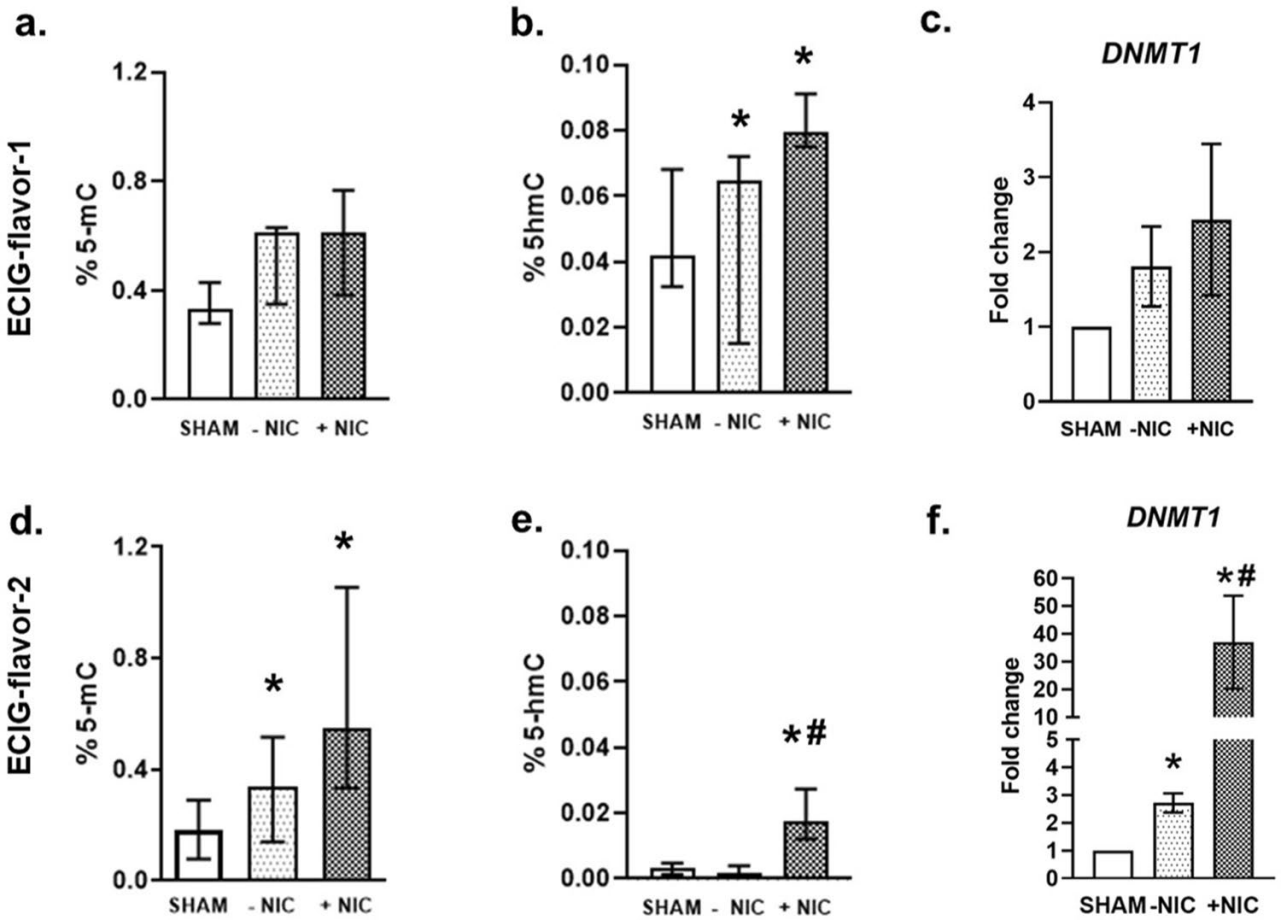
Methylation and hydroxymethylation of total DNA and transcript expression of DNA methyl transferase 1 (*DNMT1*) was assessed in the alveolar mucosa model cultured at air–liquid interface following exposure to aerosolized non-nicotinized (−NIC) and nicotinized (+NIC) electronic cigarette liquid flavor 1 (ECIG-flavor-1) (**a**–**c**) and ECIG-flavor-2 (**d**–**f**). Data are shown as percentage of 5-methylcytosine (5-mC) or 5-hydroxymethylcytosine (5-hmC). Fold changes for transcript expression were calculated relative to the corresponding sham. Actin beta (*ACTB*) was used as the reference gene. *: significantly different from sham; #: significantly different from −NIC (*p* < 0.05, Friedman followed by Wilcoxon test).

ECIG-flavor-1 (± NIC) exposed alv-ALI models exhibited an increased trend in methylation (Fig. 8a) and significantly increased hydroxymethylation (Fig. 8b) of total DNA. Expression of *DNMT1* was not significantly altered on exposure to ECIG-flavor-1 (±NIC) (Fig. 8c). On the other hand, ECIG-flavor-2 (±NIC) exposure resulted in significant increase in both methylation (±NIC) (Fig. 8d) and hydroxymethylation (only +NIC) of total DNA (Fig. 8e). Consistent with the methylation and hydroxymethylation patterns, significantly increased levels of *DNMT1* was detected and the effect was pronounced in case of +NIC flavor (> 30-fold) (Fig. 8f). Expression of *DNMT3A* and *DNMT3B* remained unchanged.

## Discussion

Risk assessment of ECIG is complex and challenging owing to the tremendous variability in delivery systems, composition of E-liquids (flavors, nicotine etc.) and vaping regimes^22,36–38^. Considering the above factors, we tested the exposure related pulmonary effect of two popular mixed fruit flavored E-liquids (±NIC) by using our multicellular bronchial- and newly developed alveolar mucosa models representing different regions of the respiratory tree. One difficulty in translating the dosimetry from our in vitro exposure system to the lung in vivo, is that the PNC as well as the PSD is likely to differ between experimental puffing system and real-world puffing and inhalation. In addition, particles of different size will deposit at different depths in the lung. Moreover, the PSD is likely to change as the aerosol travel down the respiratory tree, due to hygroscopic growth and fusion of droplets^39^. All these factors make the in vitro to in vivo dosimetry extrapolation extremely difficult. Moreover, a direct way of exposing the cells to E-smoke would be preferable compared to the indirect method we used. However, we found it difficult to perform direct exposures due to the operational restrictions of the ECIG device, as heating times more than 10 s results in overheating. Our measurements of the PSD of the E-smoke (range 0.25–3 µm) clearly suggests that it will reach the bronchial and alveolar regions of the lung. According to our measurements, the PSD of the E-smoke ranged from 0.25 µm (the lower range of the instrument) to 3 µm, with the highest PNC abundance around 0.58 µm. This is in agreement with other reports^40,41^, although some other studies reported the highest abundance at 0.02–0.3 µm^42,43^. In any case, the PSD of the E-smoke clearly suggests that the E-smoke will reach the bronchial as well the alveolar regions of the lung^44^. Hence, both lung mucosal models are relevant and useful to assess adverse effects at different levels of the respiratory tree. Regarding the alveolar model, the assembly of type 1 and type 2 pneumocytes requires further clarification including TEER value measurements. Unfortunately, there is lack of data in this regard in the literature. Meanwhile, the TEM analysis shows many characteristics of the type II pneumocytes as demonstrated by the presence of microvilli, lipid bodies, desmosomes and tight junctions.

Our detection of common respiratory irritants (like acrolein and diacetyl etc.) in the E-smoke is consistent with other reports^26,45^. Moreover, detection of 67% of the identified compounds in E-smoke as being flavoring agents or fragrances for consumer use underscores the extensive and diverse use of such additives in E-liquids. The flavoring agents maltol, ethyl maltol, ethyl vanillin, vanillin, and furaneol identified in E-smoke have been previously reported to be the most cytotoxic flavoring agents present in different flavors of E-liquids^6^. The 33% of the compounds in E-smoke without source information were predominantly aromatic hydrocarbons, these are likely byproducts of thermal degradation of which several have been shown to be potential health hazards^46,47^.

p-benzoquinone has been extensively studied in cigarette smoke and has been determined as a plausible emphysema causing factor in alveolar cells^48^. It inhibits vitamin B uptake in human lung, is genotoxic both in-vivo and ex-vivo, and imparts its toxicity on epithelial cells by disrupting microtubule networks^48,49^. p-benzoquinone was found in both the ECIG-flavors, however, information on concentrations in E-smoke and implications of health effects are limited. Therefore, p-benzoquinone in E-smoke warrants major attention. Nicotyrine, is found in much higher concentration in E-smoke than in conventional cigarette smoke^50,51^. It is formed by oxidation of nicotine in the E-liquid and may increase nicotine levels in the blood stream by irreversibly binding hepatic CYP2A6^53^. It would be of importance to study if p-benzoquinone, nicotyrine, and other flavoring chemicals identified at higher concentrations in this study are responsible for the detected toxicological response observed in bronchial and alveolar mucosa models. This would aid in implementing ECIG safety-regulation based on specific chemicals and/ or flavoring chemicals.

In general, ECIG-flavor-2 had a higher amount of flavoring and fragrance agents compared to ECIG-flavor-1. Increased numbers and concentrations of flavoring agents have been found to be directly proportional to the cytotoxicity of E-liquids^6^, suggesting ECIG-flavor-2 to be more cytotoxic compared to ECIG-flavor-1. However, ECIG-flavor-1 did have a higher levels of top cytotoxic compounds vanillin and ethyl vanillin (not detected in ECIG-flavor-2). It remains to be investigated if chemical composition of the two ECIG-flavors changes with wattage of ECIG device. Such changes in composition may obviously affect toxicity, as stated by other investigators as well^37,52^.

Exposure of the bro-ALI model to non-nicotinized E-smoke from flavor-1 did not result in any detectable changes transcript level response whereas the corresponding nicotinized flavor resulted in a strong response as reflected by higher magnitude (fold change) and number of increased transcript levels of pro-inflammatory, oxidative stress, metalloproteinases, anti-proteases, alarm anti-proteases as well as microbial-defense markers. In contrast, exposure of non-nicotinized E-smoke from flavor 2 on the bro-ALI resulted in decreased transcript expression of pro-inflammatory markers and increased anti-inflammatory marker whereas oxidative stress markers, anti-protease, alarm anti-proteases, and anti-microbial defense response markers were induced. On the other hand, exposure to nicotinized E-smoke from flavor 2 on bro-ALI exhibited mixed effect on transcript expression levels of pro-inflammatory markers, profound oxidative stress response, and corresponding induction of anti-proteases, alarm-anti-proteases and anti-microbial defense response. In general, reduced levels of the cytokines were detected in the basal media of the bronchial mucosa model exposed to both flavors (±NIC). Reduced levels of the bronchial club cell specific marker SCGB1A1 transcript and protein levels following exposure to non-nicotinized E-smoke from flavor-2 exposure indicate bronchial epithelial injury since club cells can act as stem cells. Reduced expression of *SCGB1A1* in the bro-ALI is consistent to our previous findings using diacetyl, a constituent of E-smoke^34^. The deviation between transcript and protein level expressions may be due to the time lag between transcript level signal and secretion at protein level similar to the release of pro-inflammatory mediators from intracellular stores (vesicles) or extracellular stores (matrix immobilized) and inflammatory cell recruitment^53^. In this context, it would be of interest to perform a multi-time point analysis of molecular markers both at transcript and protein level.

Similar to bro-ALI, exposure studies of E-smoke (±NIC) from the two flavors on alv-ALI model also exhibit different effects on the regulation of the investigated markers. Non-nicotinized E-smoke from one flavor (ECIG-flavor-1) caused pro-inflammation, oxidative stress, and increased metalloproteinase as a sign of tissue injury together with increased anti-proteases and anti-microbial defense response as detected by transcript expression analysis. On contrary, exposure of alv-ALI to the corresponding nicotinized E-smoke resulted in down-regulation of only the anti-oxidant *SOD3*. However, we did not detect any significant alteration of the markers at the secreted protein level in case of flavor 1 (±NIC). Reduced protein concentrations of IL1B, IL10, IL13, and TNF were detected in the basal media of alveolar models exposed to ECIG-flavor-2 (±NIC) with more pronounced effect in the (+NIC) flavor. Indications of altered barrier function, reflected by increased *TJP1* expression, in the alveolar model was observed on exposure to only nicotinized flavors. It has been reported that disruption of TJP1 is associated with epithelial barrier function following in vitro cigarette smoke exposure^54^. Improvement of pulmonary function in COPD following corticosteroid treatment is associated with increased expression of epithelial barrier function genes^55,56^. Therefore, increased expression of *TJP1* observed in our study may be regarded as a repair response to impairment of barrier function.

The overall suppressed effect of nicotinized flavors on the alv-ALI model is an interesting observation which is likely related to the immunosuppressive role of nicotine^57^. The findings also implicate that the composition of E-smoke may influence the pattern of molecular response in a lung region specific manner (i.e. bronchial or alveolar). Further, one study demonstrated that nicotine depletes S-adenosylmethionine in the alveolar but not the bronchial region rats, causing impairment of the immune response to infection^58^. This supports our finding of lung region specific action of nicotine comparing bro-ALI and alv-ALI models. However, it needs to be noted that our exposure regime in case of alv-ALI (30 puffs) was half compared to that of bro-ALI (60 puffs) due to the high sensitivity of the alv-ALI model. The high sensitivity of the alv-ALI model may be explained by the lack of the protective mucus layer in contrast to bro-ALI. Due to this we limited our statistical comparisons within each lung mucosa model and did not compare the magnitude of effects between the bronchial and alveolar mucosa models. Nevertheless, the ROS generated by separate exposures of bronchial and alveolar mucosa models to flavor 2 is consistent with transcript expression of oxidative stress markers.

While assessing and comparing the effects of E-smoke in our study with those reported in other studies, it is important to consider the exposure methods and cell lines used. In many cases, normal bronchial epithelial cells in ALI have been used but the ECIG liquid or E-smoke extract has been added in the basal medium in contrast to the E-smoke exposure in our study and in real life^16,59^. While taking into account of the nicotine concentration, majority of the studies^16,59^ used nicotine concentrations between 12 and 36 mg/mL, as compared to 3 mg/mL used in this study (relevant for Sweden and European Union). Increased proinflammatory cytokine secretion (such as IL6, CXCL8), reduced cell viability, increased oxidative stress, morphologic alteration of secretory functions, and alteration of barrier function on exposure to both high doses of non-nicotinized and nicotinized ECIG-liquid have been reported^16,59,60^. Ween et al.^61^ (2017) reported decreased TNF-α, IL6, and other pro-inflammatory cytokine levels on exposure to both +NIC (18 mg/mL) and −NIC apple flavored ECIG-liquids using THP-1 macrophages.

The effect of transcript level up-regulation of the proinflammatory, oxidative stress, extracellular matrix, alarm anti-proteases and anti-microbial response markers following exposure to ECIG-flavor-1 (−NIC) compared to corresponding +NIC flavor on the alv-ALI model are striking. However, none of the markers assessed at protein level were significantly altered in case of alv-ALI model exposed to ECIG-flavor-1 (±NIC). Nicotine acts both as a pro- and anti-inflammatory bioactive molecule^3^. Anti-inflammatory effects of nicotine have been demonstrated in human bronchial epithelial cells following comparison with cigarette smoke condensate, nicotine alone, and pre-treatment with nicotine^3,62^. It has been reported that nicotine can suppress secretion of proinflammatory molecules like CXCL8, IL6, TNF following exposure to cigarette smoke extract and lipopolysaccharide^62^. Anti-inflammatory effects of nicotine have also been shown by nicotine induced inhibition of acute lung injury in mice^3,63^. It is plausible that the anti-inflammatory effect of nicotine may be modulated through the stimulation of nicotinic receptors present in the lung and this may be cell-type specific. Data supporting anti-inflammatory effects of nicotinic receptor agonists by reducing acute lung injury are also available^3,64–66^. In contrast, pro-inflammatory effects of nicotine in different experimental models of the lung epithelium are also reported^3,67,68^. In our experimental set up, the observed effects on bro-ALI and alv-ALI seem to be related to both the flavors and the nicotine content of the E-smoke. It would be of interest to study the different doses of nicotine using the same flavor composition to understand the anti- or pro-inflammatory effects on bronchial and alveolar mucosa models.

E-smoke from flavor-2 seem to be a more potent modifier of methylation and hydroxymethylation compared to flavor-1. This is also supported by the transcript up-regulation of DNA methytransferase 1. The effects were more pronounced in the nicotinized flavors. Both methylation and hydroxymethylation are considered as important mechanisms of epigenetic modifications that may result in altered gene regulation^69^. Overall, the findings are in agreement with the chemical composition analysis predicting E-smoke from flavor-2 to have higher toxic potential compared to that of flavor-1. Taken together, these observations suggest a flavor specific mode of action of E-smoke which may be altered with nicotinization. Recently, Muthumalage et al.^60^ (2020) compared the composition of E-smoke from counterfeit patient cartridges, cannabidiol (CBD)-cartridges, and medical grade cartridges. A varying constitution of terpenes, silicon compounds, pesticide, flavor additives, cannabinoids, plasticizers, humectants, vitamin and conjugates were detected. Therefore, assessment of E-smoke of different flavors (±nicotine) plausibly at different power settings of ECIG device is warranted to create a framework of ECIG risk assessment.

Findings of our study is consistent with other data reporting the pro-inflammatory reaction and oxidative stress effects of E-smoke^4,70^. Our data indicates the plausible effect of E-smoke on the protease/anti-protease balance together with anti-microbial defense response as an important target in both bronchial and alveolar mucosa models. The pattern and extent of effects may be influenced by the lung region, flavor and/ or nicotine content. Proteases and anti-proteases are secreted from the respiratory epithelium and play an important role in maintaining respiratory homeostasis^71^. Altered protease/anti-protease balance are known to play key role in the development of chronic lung diseases like emphysema and COPD^71^. Respiratory anti-proteases are comprised of TIMPs, serine protease inhibitors (serpins), trappins-2/elafin and SLPI with unique target substrates, cellular sources, and anti-protease functions^71^. Increased MMP9 and its main inhibitor TIMP1^71^ was detected following E-smoke exposures. Elafin (PI3) and SLPI, produced by respiratory epithelia, club cells, type II cells etc. are known as alarm anti-proteases that inhibits neutrophil elastase and is also known to exhibit broad anti-microbial, anti-inflammatory, innate and adaptive immune response, tissue remodeling and wound healing effects^72,73^. Secretion of alarm anti-proteases are influenced by proinflammatory molecules such as IL1 and TNF^74,75^. DEFB4A is a multifunctional peptide with anti-microbial activity together with roles in innate and adaptive immunity, inflammatory and anti-inflammatory response, immunomodulation, and wound repairing^76^. It is expressed throughout the respiratory epithelium^76^. Altered expression of DEFB4A has been associated with several respiratory diseases like asthma, COPD, pulmonary fibrosis, pneumonia, tuberculosis, and rhinitis^76^. Elevated levels of DEFB4A have been reported in association with cystic fibrosis and COPD whereas reduced levels have been detected in case of asthma via TH2 type response^77^. It is plausible that altered regulation of *DEFB4A* following E-smoke exposure may affect response to infection. Chronic exposure to E-smoke have been implicated to higher susceptibility for infection in mouse models^18^. Our results with the bronchial and alveolar mucosa models indicate that the pattern of regulation of *PI3, SLPI*, and *DEFB4A* depends on the flavor, nicotine content of E-liquid as well as the lung region. Consistent with our findings with ECIG-flavor-2 in the bronchial model, decreased SCGB1A1, increased SLPI, and increased PI3 levels were detected in the sputum from COPD patients compared to smokers without COPD and/or never smokers^78^. However, we detected increased transcript levels of *DEFB4A* in the bronchial mucosa model whereas DEFB4A was suppressed in the COPD patients^78^.

To summarize, in our chemical analysis of the E-smoke from the two flavors, we detected a number of cytotoxic chemicals as well as significant differences in composition. In the particle characterization we observed a marked increase in PNC with the wattage applied and a slight increase with nicotine present. These findings suggest that the choice of E-liquid and delivery device/wattage will affect the composition and dosimetry during vaping, and hence also potential health effects. The role of compounds like p-benzoquinone, nicotyrine, and flavoring agents detected in higher concentrations warrants independent studies to evaluate their role in ECIG toxicity. The different patterns of molecular response in the bronchial and alveolar mucosa models suggest lung region specific effects including the role of nicotine. Further, apart from inflammatory and oxidative stress response, regulation of alarm-antiproteases and anti-microbial defense response factors as well as barrier function and epigenetic modifications following E-smoke exposure may play an important role in imparting toxic effects. To conclude, our study identifies the need of multi-disciplinary approach for comprehensive safety profiling of ECIG products.

## Supplementary information

Supplementary Figures.

Supplementary Legends.
