# Supplementary material for: Addressing the challenges of E-cigarette safety profiling by assessment of pulmonary toxicological response in bronchial and alveolar mucosa models
Source: Sci Rep. 2020 Nov 24;10:20460. doi: 10.1038/s41598-020-77452-w (PMC7686373; doi:10.1038/s41598-020-77452-w)

Supplementary Material

Addressing the challenges of E-cigarette safety profiling by assessment of pulmonary toxicological response in bronchial and alveolar mucosa models

Koustav Ganguly[1#], Axel Nordström[1], Tania A. Thimraj[1], Mizanur Rahman[1], Malin Ramström[1], Shanzina I. Sompa[1], Elizabeth Z. Lin[2], Fiona O'Brien[2], Jeremy Koelmel[2], Lena Ernstgård[1], Gunnar Johanson[1], Krystal J. Godri Pollitt[2], Lena Palmberg[1*], Swapna Upadhyay[1*#]

[1]Unit of Integrative Toxicology, Institute of Environmental Medicine (IMM), Karolinska Institutet, Sweden

[2]Department of Environmental Health Sciences, School of Public Health, Yale University, New Haven, CT, US.

[#]Correspondence:

Koustav Ganguly

Unit of Integrative Toxicology

Institute of Environmental Medicine (IMM)

Karolinska Institutet

Email: koustav.ganguly@ki.se

Phone:+46-707646787

Swapna Upadhyay

Unit of Integrative Toxicology

Institute of Environmental Medicine (IMM)

Karolinska Institutet

Email: swapna.upadhyay@ki.se

Phone:+46-736989581

Materials and Methods

E-smoke generation

Third generation electronic nicotine delivery systems (open system) or electronic cigarettes (ECIG) with refillable and exchangeable tank (2ml) options purchased from the same manufacturer with identical specifications were used to generate the ECIG aerosol (E-smoke). Two popular mixed fruit sweet flavored (E-liquid1: Raspberry, orange, lemon and Lime; E-liquid 2: Ripe strawberry, sweet apples and tart kiwi) E-liquids (±nicotine) from the same manufacturer were used for experimental purposes. E-liquids were purchased from the same manufacturer in order to maintain the consistency of the basic constituents (glycerol/propylene glycol ratio: 70:30). The lowest nicotine concentration (3mg/mL) available on the Swedish market was used. The choice of E-liquids and ECIG device was based on adolescence preference for sweet fruity flavors[1], usage of low nicotine concentrations, and popularity of the ECIG open-system. The ECIG devices used could support a coil resistance range of 0.1-3.5Ω and the output wattage could be adjusted to 1W-75W. The temperature of the instrument could be adjusted between 100-315°C. The power setting 40W was the default setting of the instrument and anything above 50W is considered as advanced vaping. Atomizers/replaceable heating coils with 0.3Ω was used based on the easy availability in the vaping stores. We used separate ECIG devices and separate tanks for the nicotinized (+NIC) and non-nicotinized (-NIC) E-liquids for each flavor to avoid contamination. The E-liquid tanks were refilled to avoid dry puffs and cleaned prior to each experiment.

A vaping regime representing a low intensity user for a short-term repeated exposure for one day was mimicked in this study considering 40ml puff volume, aerosolization of E-liquid at 40W, 3 second (s) puff duration, and a total of 10 puffs per vaping session for exposing the human alveolar and bronchial mucosa models. This was repeated 6 times during a day with an interval of 1 hour (h) between each session, leading to a total of 60 puffs. The alveolar models did not survive the 6 repeats and therefore were exposed thrice resulting into 30 puffs.

Particle number concentration and size distribution

Particle number concentration (PNC; counts per cm^3) and particle size distribution (range: 0.25-32µm) of the E-smoke was measured for both E-liquid 1 and 2 (+NIC and -NIC) using the portable laser spectrometer (LAS) [model Mini-LAS 11R; GRIMM, Aerosol Technik GmbH and Co. KG, Germany). The instrument output of PNC was counts per litre. The data was analyzed using the manufacturer provided GRIMM Windows 1178 software (LabView Software 1.178 V4.0). E-smoke was generated at three different power settings (25, 40 and 55W) representing low, medium and high wattage for measuring the particle concentration and size distribution.

Using a preheated air-tight glass syringe, 40ml (i.e. representing 1 puff) of aerosolized (3s, representing 1 puff duration) E-liquid was collected. The glass syringe was pre-heated before collection of E-liquid vapor to avoid condensation and agglomeration of particles. A vapor volume of 2ml was injected into an air-tight 32L plastic container where the suction head of the LAS was inserted. Measurements were performed at an interval of 6s for 1.5 minutes (min) to ensure that the peak particle count was detected. Afterwards, the vapor was emptied inside a fume hood using compressed air. Prior to each injection, background air particle concentration was measured inside the plastic box, which was subtracted from the average particle levels obtained after each vapor injection. The mean PNC from 15 vapor injections was measured for each E-smoke combination. The results are represented as a standard error of mean of 15 measurements and the data was normally distributed.

Chemical characterization of E-smoke

Eleven compounds commonly reported to be present in E-smoke were selected for detection using gas chromatography with flame ionization detector (GC-FID). The compounds were: 1-pentanol, 2,3- pentadione, acetoin, acetic acid, acetone, acrolein, crotonaldehyde, diacetyl, methanol, propionalaldehyde and toulene. In addition, nicotine was also detected. After collection of 40 ml E-smoke (in the pre-heated glass syringe) produced by a 3s puff at 40W from ECIG-liquid-flavor-1 (±NIC), 2 ml of the E-smoke was immediately transferred by a glass syringe to stainless steel adsorption tubes filled with Tenax or Carbotrap (20/40 Mesh) and Carboseive SIII (60/80) (all from Supelco Park, Bellefonte, USA). Triplicate samples were collected. Samples were desorbed (Automated Thermal Desorption system ATD-400, Perkin Elmer Ltd. Beaconsfield, England) and analysed by gas chromatography (GC, Clarius 580, Perkin Elmer, USA). The thermal desorption settings were: desorption oven 330˚C, desorption time 5 min, valve 200 ˚C, trap low -30˚C, high 300˚C, isothermal 2 min, line 200˚C and pressure 3.6 psi (about 0.5 ml/min). The GC was equipped with a 30 m Rtx-VMS capillary column (i.d. 0.25 mm, coating 1.40 µm, no 199159, Restek, Bellefonte, USA) and a flame ionization detector. The column temperature was initially kept at 55˚C for 1 min, then raised to 250˚C by 11˚C/min and kept for 15 min. The temperature of the detector was 260˚C. Both samples with and without nicotine were analysed at Tenax and Carbotrap/Carboseive tubes. The peaks in the aerosol samples were compared to chromatograms of known compounds (reference compounds) that had been previously analysed.

For suspect screening of select analytes, custom fabricated polydimethylsiloxane (PDMS) sorbent bars which were placed inside the exposure chamber for 15 min. 40ml E-smoke (in the pre-heated glass syringe) produced by a 3s puff at 40W was injected into the exposure chamber. Three replicates were collected for each E-Smoke flavor (±NIC). PDMS sorbent bars

were cleaned following previously described methods[2]. Following the exposure sampling period, PDMS sorbent bars were transferred to air-tight amber glass vials and stored at -20 °C until analysis at the Yale School of Public Health. Immediately prior to analysis, PDMS sorbent bars loaded with an internal standard mixture. PDMS sorbent bars were then placed into pre-cleaned glass autosampler tubes (Gerstel, Linthicum, MD, USA) on a temperature controlled autosampler tray maintained at 10 ºC (MéCour, Groveland, MA, USA). For sample analysis, an autosampler tube was transferred into a thermal desorption unit (TDU; Gerstel, Linthicum, MD, USA). The TDU was initially held at 30°C for 1.1 min and then ramped at 720 °C per minute to 280 °C (5 min hold) under a flow rate of 350 mL/min of helium gas (99.999%). Extracted analytes were cyro-focused to -90 ºC on a 2mm, glass wool deactivated liner in a cooled injection system (Gerstel, Linthicum, MD, USA) cooled to -90 ºC. The transfer line between the TDU and cooled liner was maintained at 250 °C. Analyses were directly transferred to the GC column (TG-5SILMS, 30m x 0.25mm x 0.25 µm). The carrier gas flow (helium) was set to 1.4 mL/min and the GC oven was held at 70 °C for one minute and then ramped at 7 °C/min to 300 °C. The final temperature was held for 4.0 min for a total run-time of 37.86 minutes. During the analysis, full-scan electron ionization (EI) mass spectra (m/z 53.4 – 800) was recorded at an acquisition rate of 4 Hz. QCs and blanks (laboratory and transport) which were run every 10 samples. The suspect screening Thermo Deconvolution Plugin (Thermo Fisher Scientific, Waltham, MA) was used for suspect screening using the NIST 17 and Thermo spectral libraries. Alkanes were used to calculate Kovat's retention index for all compounds. The compounds were searched against the NIST 17 and Thermo libraries. Higher confidence was obtained for tentative identifications using the following filters: retention index total maximum deviation: 50, and percent deviation: 1.5%; reverse search index greater than 600, reverse high-resolution filter greater than 75, and total score greater than 75. The high-resolution filter calculates the formula of fragments using only the atoms contained in the molecular formula, and higher scores indicate a higher portion of fragments which have exact masses which can be predicted using formulas obtained from these atoms in the molecular formula. Reverse searches indicate that only fragments from the library were considered in the scoring algorithm (reducing penalization from co-eluting compounds and background).

The resulting identifications were aligned, and gap filled across sample using a 0.03-min tolerance for alignment. The aligned and gap filled peak heights were exported and converted into TIC values (total summed deconvoluted EI spectrum) using the ratio of the average heights to the average TIC (average TIC being provided by the deconvolution software). The compound list was then blank filtered, where features were retained if the maximum of samples was greater than 10x the average of the blank signal. Relevant sources/categories of the

identified compounds in the final annotated dataset were determined using the CPDat database.

Cytotoxicity assessment following exposure

Cell viability

Trypan blue staining: Trypan blue staining was used to estimate NCI-H441 cell viability and evaluate our newly established the alveolar mucosa model quality at 24h, 4 days, 1 week and 2 weeks at ALI condition. Cells were stained with 200 µl of 1:1 in PBS diluted 0.4% trypan blue solution for 1 min. After washing with PBS, viability was evaluated using bright-field microscopy.

Propidium iodide staining:

To access cell viability, NCI-H441 cells were also stained with propidium iodide (BD bioscience, cat-556463) according to manufacturer protocol, and the retention of the propidium iodide was ascertained by flow cytometry.

Total reactive oxygen species (ROS) measurement:

Total ROS was measured in bronchial (2 donors and 3 replicates/donor) and alveolar mucosa (2 passages; 3-4 replicates/passage) models following exposure to 3 vaping sessions each (10 puffs/session ie. 30 puffs in total) of E-smoke generated from ECIG-flavor-2 at 40W. This was performed as a control experiment to assess if bronchial and alveolar models also react differently under same exposure conditions. Total ROS was measured in the cells using flow cytometry 2 hours after the exposure. Cells were stained with CellROX green reagent (Catalog number: C10444; Thermofisher Scientific) for 30 minutes in incubator. Following incubation, cells were washed, collected and ROS were measured. The level of total ROS is presented as median fluorescent intensity (MFI).

Evaluation of tight junction: In order to determine the integrity of the alveolar mucosa model, the transepithelial electrical resistance (TEER) was measured with an EVOM Volt Ohm Meter equipped with an EndOhm (World Precision Instruments, USA). TEER values were measured at 24h, 4 days, 1 week and 2 weeks ALI condition with STX01 electrodes connected to a Millicell ERS voltohm meter (Millipore, Carrigtwohill, Ireland). TEER values were corrected for the background value contributed by contributed by growth medium using TEER of polyester transwell filter membranes without cells filled with PBS considering as a blank insert (without cells). For cells cultured under ALI conditions, fluid volumes were adjusted to 500 µl apically and 1ml at basal chamber with pre-warmed medium. TEER values were obtained from at least 5 inserts at each time points. TEER was calculated as follows: (TERR value of inserts with

cells- TEER value of inserts without cells or blank inserts) x surface area of transwell membrane (0.9 cm^2). Additionally, integrity of the model was assessed using immunofluorescence staining of zona occluding (ZO-1).

Histological analysis:

Histological analysis

For histological analysis of co-cultured alveolar mucosa model cultured at air-liquid-interface (alv-ALI), membranes were cut from insert and fixed in 4 % formalin overnight. After dehydration in graded ethanol, the membranes were embedded in paraffin and sectioned at 5 µm thickness. The sections were stained with Hematoxylin and Eosin or Periodic acid–Schiff. The staining profiles in the sections were captured and visualized using BX50 light microscope (Olympus Optical Co., Tokyo, Japan).

Confocal microscopy

First alv-ALI models were fixed by adding 1ml of 4 % formalin of paraformaldehyde on both (apical and basal) side of the inserts and incubated for 30 minutes. After that model membranes were washed twice using 1X by PBS and then membranes were incubated with blocking buffer (PBS+ 0.1% Triton X+goat serum) for maximum 15 minutes. Then the membranes were stained with primary antibodies: rabbit anti-human ZO1 (ab96587, 1:100) rabbit anti-human pro-surfactant protein C (SP-C) polyclonal antibody (ab90716: 5 µg/ml.), LysoTracker Green DND-25 (L7526, working concentration 1µM) and rabbit anti-human epithelial sodium channel (aENaC) polyclonal antibody (2 µg/mL; PA1-920A) separately for overnight at 4°C. On Day 2 secondary antibodies: Alexa Fluor 488-conjugated goat anti-mouse IgG (1:500; ab150077), Alexa Fluor™555 Phalloidin (A34055: 1:100) were added, except for LysoTracker Green DND-25 (no secondary antibody required). Finally, membranes were mounted on microscope slides with DAPI (ab104139). Images were captured and visualized using a LSM700 confocal microscope (Zeiss, Germany).

References:

[1] Zare S, Nemati M, Zheng Y. A systematic review of consumer preference for e-cigarette attributes: Flavor, nicotine strength, and type. PLoS One [Internet]. 2018 Mar 15;13(3):e0194145–e0194145. Available from: https://www.ncbi.nlm.nih.gov/pubmed/29543907

[2] Lin EZ, Esenther S, Mascelloni M, Irfan F, Pollitt KJG; The Fresh Air Wristband: A Wearable Air Pollutant Sampler; Environ. Sci. Technol. Lett. 2020,; Publication Date: January 28, 2020; https://doi.org/10.1021/acs.estlett.9b00800

Supplementary table ST1: Forward and reverse primer sequences of aquaporin 5 (*AQP5*), defensin beta 4A (*DEFB4A*), peptidase inhibitor 3 (*PI3*), and surfactant proteins (*SP*) *A,B,C* ; tight junction protein 1(TJP1), DNA methyl transferases (*DNMT1, 3A, 3B*) and claudins (*CLDN5,7*) used for qRT-PCR analysis.

**Gene Name**	**Nucleotide sequence** (5′→3′)
AQP5 (forward)	ACTGGGTTTTCTGGGTAGGG
AQP5 (reverse)	GTGGTCAGCTCCATGGTCTT
*DEFB4A (*forward)	CTCCTTCCTCTCTCTGTGTGTCCC
*DEFB4A (*reverse)	CGAGTGAGTTGTCCATTGGGTTC
*PI3 (*forward)	CGGAATTCATGTAGGAAAACTCTTCGGACA
*PI3 (*reverse)	CGCGGATCCGAATGAGATGGCAAAGAACA
SPA (forward)	AGGAGCTCCAAGCCACACT
SPA (reverse)	TTCCTCTGGATTCCTTGGG
SPB (forward)	TGGGAGCCGATGACCTATG
SPB (reverse)	GCCTCCTTGGCCATCTTGT
SPC (forward)	ATCGGCTCCACTGGCCTCGT
SPC (reverse)	AGTAGAGCGGCACCTCGCCA
DNMT1, 3A, 3B; TJP1; CLDN ,5,7	Predesigned primer pairs from Sigma-Aldrich (KiCqStart™ Primers) https://www.kicqstart-primers-sigmaaldrich.com/KiCqStartPrimers.php?domain=STANDARD

Supplementary table ST2: Particle number concentration (PNC; counts per cm^3) of non-nicotinized (-NIC) and nicotinized (+NIC) electronic cigarette (ECIG) liquid flavors 1 and 2 aerosolized at 25W, 40W and 55W. Data are represented as mean ± standard error of mean (SEM) of n=15 measurements. significance: p<0.05

**ECIG device power**	**ECIG-flavor-1**		**ECIG-flavor-2**	
	**-NIC**	**+NIC**	**-NIC**	**+NIC**
**25W**	8.12±0.56 ×10E04	9.91±0.57 ×10E04 $^{£}$	8.17±0.51 ×10E04	9.02±0.41 ×10E04
**40W**	13.67±0.86 ×10E04*	11.86±0.83 ×10E04$^{£\$}$	12.80±0.57 ×10E04*	14.94±0.99 ×10E04$^{*\$}$
**55W**	13.58±1.03 ×10E04*	14.02±1.01×10E04$^{*\$}$	15.65±0.86 ×10E04$^{*\#}$	17.45±0.92 ×10E04$^{*\$£}$

Significance:
***:** significant within same flavor (-NIC or +NIC) compared to 25W (p<0.001)
#: significant within same flavor (-NIC or +NIC) compared to 40W (p<0.001)
£: significant within same flavor -NIC versus +NIC compared within same wattage (p<0.05)
$: significant between different flavors (-NIC or +NIC) compared within same wattage (p<0.05)

Supplementary table ST3: Detection of commonly reported twelve compounds present in electronic cigarette (ECIG) liquid aerosol using gas chromatography with flame ionization detector (GC-FID) in aerosolized non-nicotinized (-NIC) and nicotinized (+NIC) ECIG-flavor-1. √**:** detected, **×**: undetected

	**Compound**	**Cas No**	**-NIC**	**+NIC**	**Retention time** (minutes)
**1**	1-pentanol	71-41-0	√	√	16
**2**	2,3- pentadione	600-14-6	√	√	14.7
**3**	Acetoin	513-86-0	×	√	13
**4**	Acetic acid	64-19-7	√	√	15.6
**5**	Acetone	67-64-1	√	√	13.0
**6**	Acrolein	107-02-8	√	√	10.9
**7**	Crotonaldehyde	123-73-9	×	√	14.3
**8**	Diacetyl	431-03-8	√	√	12.4
**9**	Methanol	67-56-1	√	√	9.4
**10**	Nicotine	54-11-5	×	√	30.6
**11**	Propionaldehyde	123-38-6	×	√	10.8
**12**	Toluene	108-88-3	√	√	15.3

Supplementary figure S1: Particle number concentration (PNC; counts/cm^3) of electronic cigarette (ECIG) aerosol from flavors 1 and 2, non-nicotinized (-NIC) and nicotinized (+NIC), generated at three different power settings (wattages; 25W, 40W and 55W) of the delivery. **a.** ECIG-flavor-1, **b.** ECIG-flavor-2; Data are represented as mean ± standard error of mean; n=15 measurements; *: significantly different from 25W ($p<0.001$); #: significantly different from 40W ($p<0.001$); £: significantly different from -NIC ($p<0.05$); $: significant difference between flavors 1 and 2 with same wattage and nicotine content ($p<0.05$)

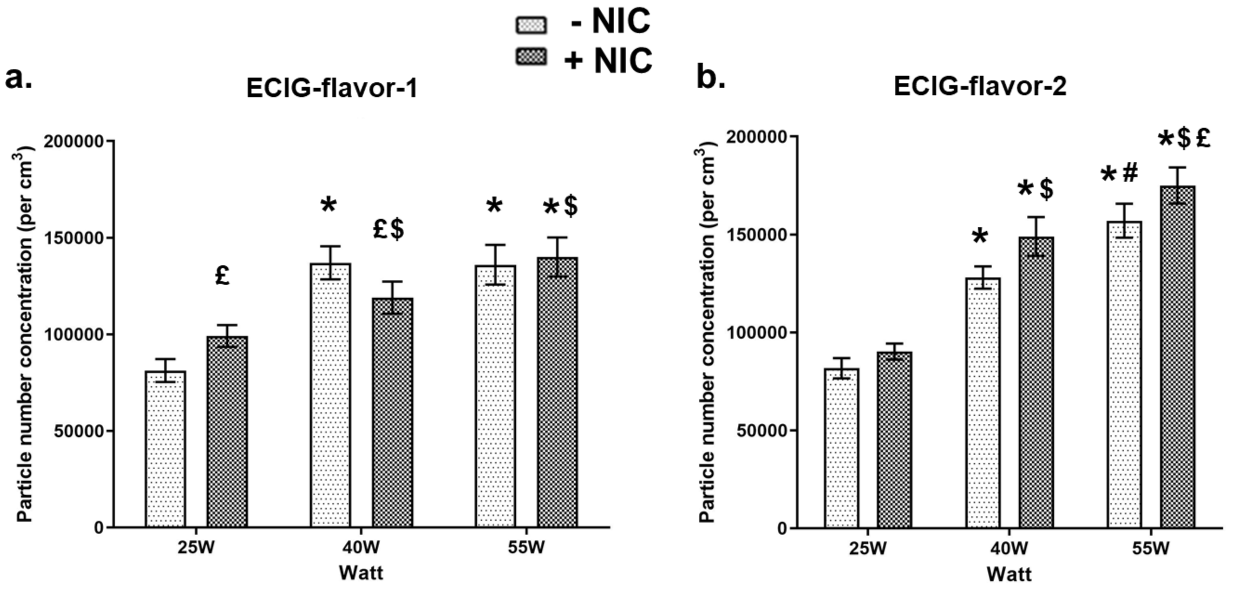

Supplementary figure S2: Particle size distribution (PSD) of electronic cigarette (ECIG) aerosol from ECIG-flavors 1 (**a-c**) and 2 (**d-f**) [±nicotine (NIC)] at low (25W), medium (40W) and high (55W) power settings of the ECIG device. Data are represented as mean ± SEM of n=15 measurements/flavor (±NIC). Particle number concentrations (PNC) is used as the instrument output (counts per litre).

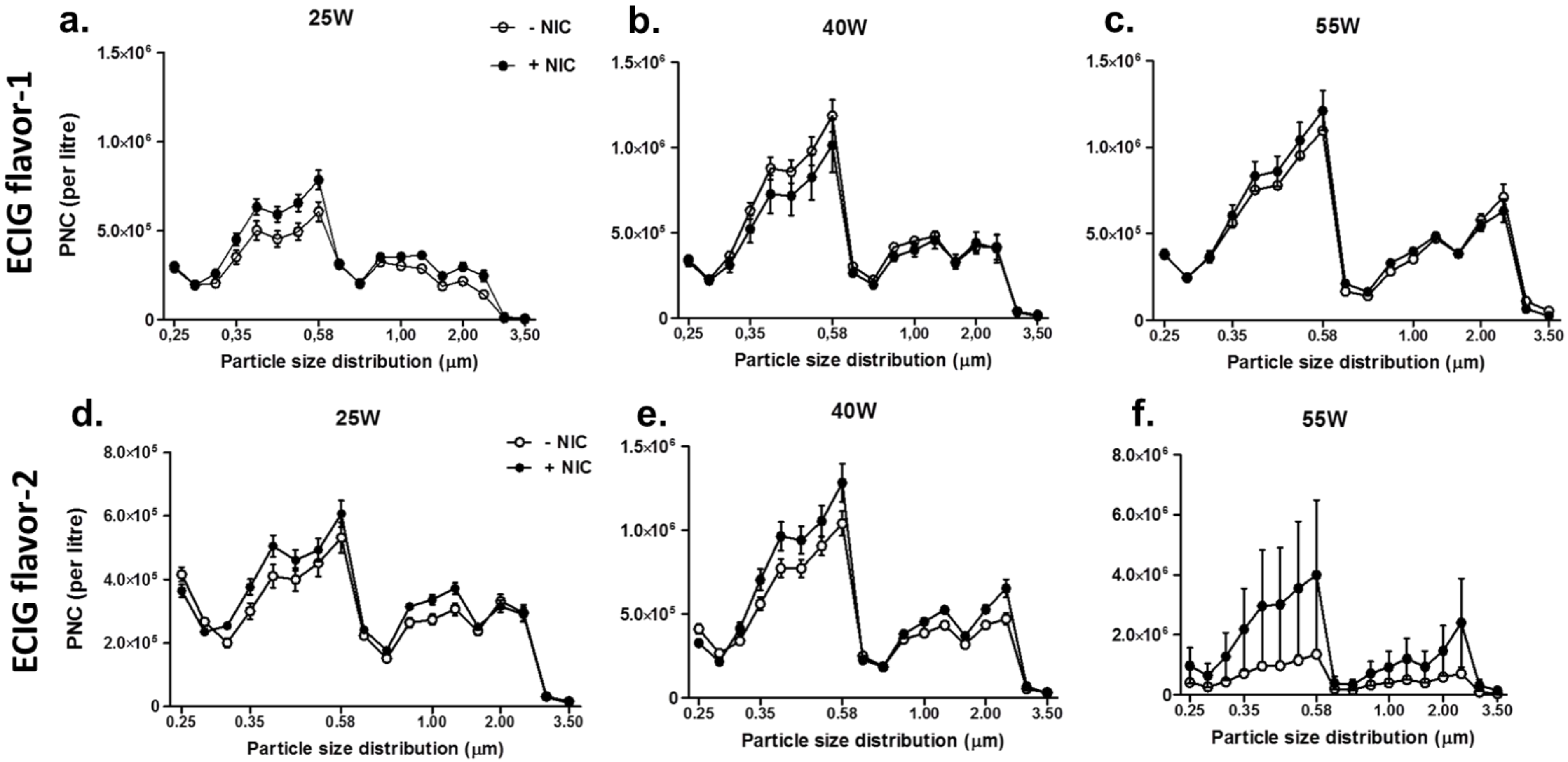

Supplementary figure S3: **a.** Transepithelial electrical resistance (**TEER**), **b.** light microscopy following Hematoxylin and Eosin staining (representative of n=5 observations), and **c-f**. quantitative real time polymerase chain reaction of surfactant proteins *(SP) A.B,C* and aquaporin 5 (*AQP5*) was performed to characterize the alveolar mucosa model cultured at air-liquid-interface. Fold changes have been calculated relative to the models at day 1. Three different passages (51-53) and two technical replicates of each passage have been used for NCl-H441 cells to develop alv-ALI models. (p<0.05, Friedman followed by Wilcoxon test). *: significant compared to day1.

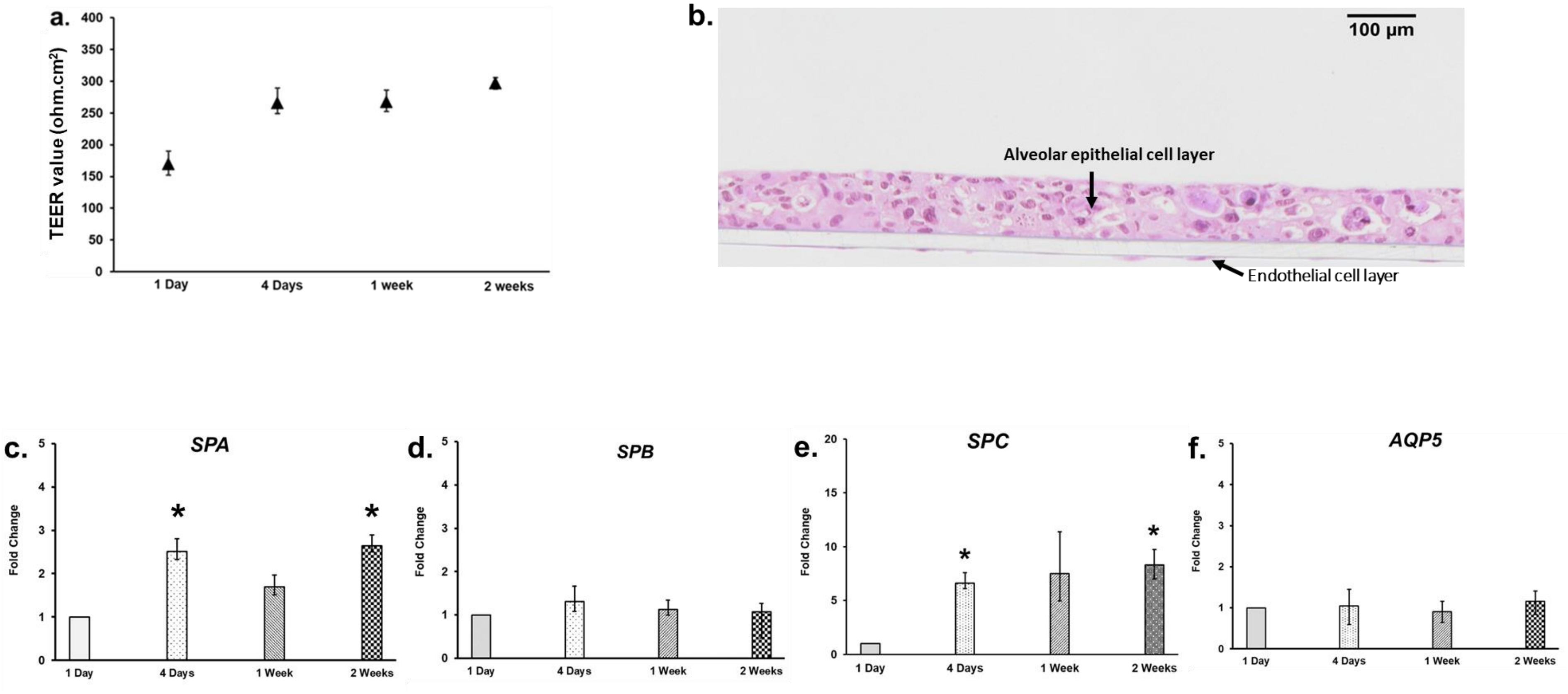

Supplementary figure S4: Transcript expression analysis of surfactant proteins *(SP) A.B,C* and aquaporin 5 (*AQP5*) by quantitative real time polymerase chain reaction in the alveolar mucosa model cultured at air-liquid interface (alv-ALI) following exposure to aerosolized non-nicotinized (-NIC) and nicotinized (+NIC) electronic cigarette liquid flavors (ECIG-flavor) 1 and 2. Actin beta (*ACTB*) was used as the reference gene. Fold changes have been calculated relative to the corresponding sham. Three different passages (51-53) and two technical replicates of each passage have been used for NCl-H441 cells to develop alv-ALI models. ($p<0.05$, Friedman followed by Wilcoxon test). *: significant compared to corresponding sham.

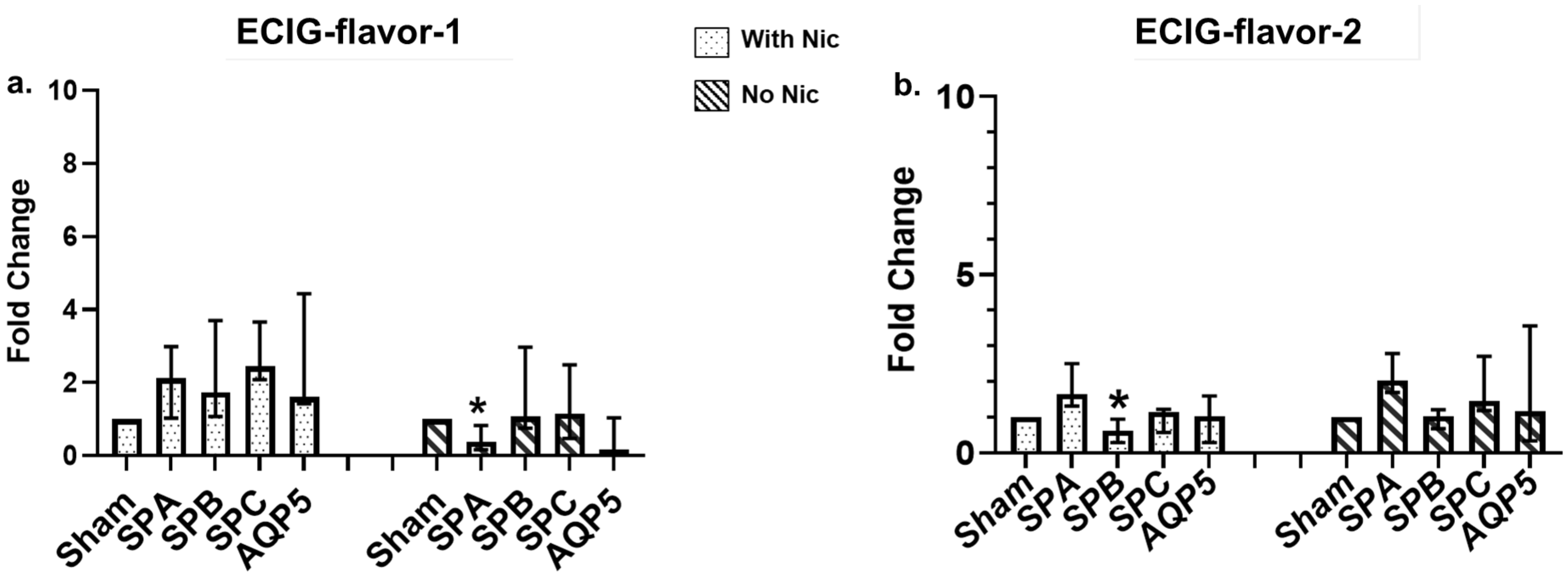

Supplementary figure S5: Transcript expression analysis of tight junction protein 1 (*TJP1*) alternatively known as zona occluding 1 (ZO1) as a marker of epithelial barrier function by quantitative real time polymerase chain reaction in the alveolar mucosa model cultured at air-liquid interface (alv-ALI) following exposure to aerosolized non-nicotinized (-NIC) and nicotinized (+NIC) electronic cigarette liquid flavors (ECIG-flavor) 1 and 2. Actin beta (*ACTB*) was used as the reference gene. Fold changes have been calculated relative to the corresponding sham. Three different passages (51-53) and two technical replicates of each passage have been used for NCl-H441 cells to develop alv-ALI models. ($p<0.05$, Friedman followed by Wilcoxon test). *: significant compared to corresponding sham; #: significant compared to corresponding -NIC.

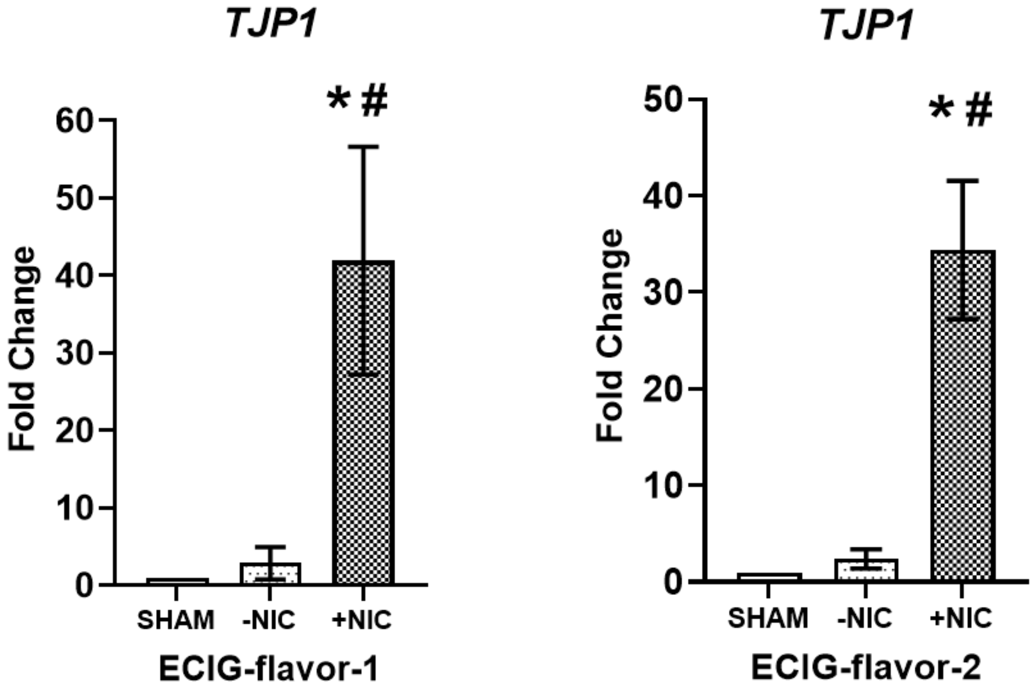

Supplementary figure S6: Total reactive oxygen species (ROS) was measured in bronchial (2 donors and 3 replicates/donor) and alveolar mucosa (two passages; 3-4 replicates/passage) models cultured at air-liquid-interface (ALI) following exposure to 3 vaping sessions each (10 puffs/session ie. 30 puffs in total) of E-smoke generated from ECIG-flavor-2 at 40W. The level of ROS in the cells is presented as median fluorescent intensity (MFI). **bro-ALI:** bronchial model at ALI; **alv-ALI:** alveolar model at ALI; $p<0.05$, Friedman followed by Wilcoxon test). *: significant compared to corresponding sham; #: significant compared to corresponding -NIC.

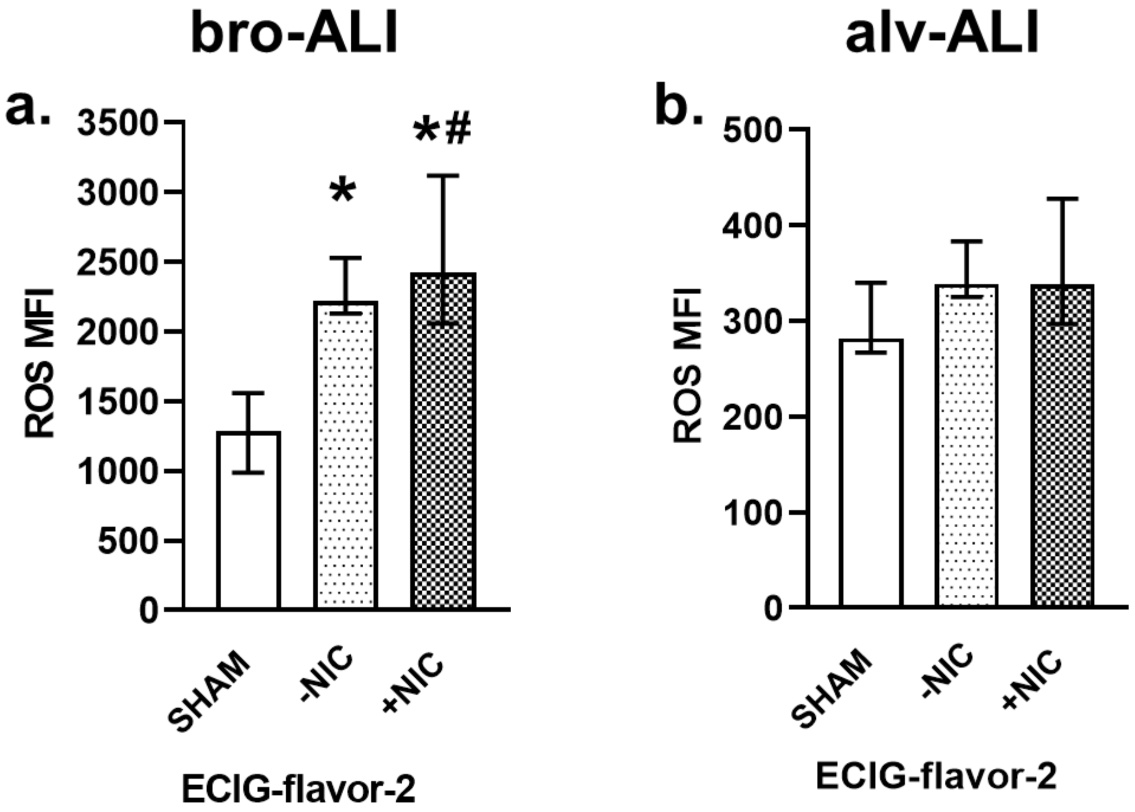

Supplement: Supplementary file 2 — Supplementary Legends. [file 41598_2020_77452_MOESM2_ESM.pdf]
